# Multimodal Image Guidance in Subthalamic Deep Brain Stimulation for Parkinson's Disease

**DOI:** 10.1002/ana.78206

**Published:** 2026-04-17

**Authors:** Patricia Zvarova, Christina van der Linden, Ningfei Li, Konstantin Butenko, Thea Berger, Garance M. Meyer, Ilkem Aysu Sahin, Lukas L. Goede, Bahne H. Bahners, Barbara Hollunder, Till A. Dembek, Andrew R. Pines, Martin Reich, Jens Volkmann, Vincent J. J. Odekerken, Rob M. A. de Bie, Xin Xu, Zhipei Ling, Chen Yao, Andrea A. Kühn, Surjo R. Soekadar, Kerstin Ritter, Michael T. Barbe, Veerle Visser‐Vandewalle, Michael D. Fox, Jan Niklas Petry‐Schmelzer, Nanditha Rajamani, Andreas Horn

**Affiliations:** ^1^ Movement Disorder and Neuromodulation Unit, Department of Neurology Charité‐Universitätsmedizin Berlin, Corporate Member of Freie Universität Berlin and Humboldt‐Universität zu Berlin Berlin Germany; ^2^ Einstein Center for Neurosciences Berlin, Charité – Universitätsmedizin Berlin Berlin Germany; ^3^ Center for Brain Circuit Therapeutics, Department of Neurology, Brigham and Women's Hospital Harvard Medical School Boston MA USA; ^4^ Network Stimulation Institute, Department of Stereotactic and Functional Neurosurgery University Hospital Cologne Cologne Germany; ^5^ Department of Neurology, Faculty of Medicine and University Hospital Cologne University of Cologne Cologne Germany; ^6^ Department of Neurology, Center for Movement Disorders and Neuromodulation, Medical Faculty and University Hospital Düsseldorf Heinrich Heine University Düsseldorf Düsseldorf Germany; ^7^ Institute of Clinical Neuroscience and Medical Psychology Medical Faculty and University Hospital Düsseldorf, Heinrich Heine University Düsseldorf Düsseldorf Germany; ^8^ Berlin School of Mind and Brain Humboldt‐Universität zu Berlin Berlin Germany; ^9^ Department of Psychiatry, Brigham & Women's Hospital Harvard Medical School Boston MA USA; ^10^ Department of Neurology University Clinic of Würzburg Würzburg Germany; ^11^ Department of Neurology Amsterdam University Medical Center, University of Amsterdam Amsterdam The Netherlands; ^12^ Department of Neurosurgery Chinese PLA General Hospital Beijing China; ^13^ Department of Neurosurgery Hainan Hospital of Chinese PLA General Hospital Sanya China; ^14^ Department of Neurosurgery, The National Key Clinic Specialty, Shenzhen Key Laboratory of Neurosurgery the First Affiliated Hospital of Shenzhen University, Shenzhen Second People's Hospital Shenzhen China; ^15^ Bernstein Center for Computational Neuroscience Berlin Berlin Germany; ^16^ NeuroCure Clinical Research Centre Charité – Universitätsmedizin Berlin, corporate member of Freie Universität Berlin and Humboldt‐Universität zu Berlin Berlin Germany; ^17^ Clinical Neurotechnology Laboratory, Department of Psychiatry and Neurosciences (CCM) Charité ‐ Universitätsmedizin Berlin Berlin Germany; ^18^ Berlin Center for Advanced Neuroimaging (BCAN) Charité — Universitätsmedizin Berlin Berlin Germany; ^19^ Hertie Institute for AI in Brain Health University of Tübingen Tübingen Germany; ^20^ Department of Stereotactic and Functional Neurosurgery Faculty of Medicine and University Hospital Cologne, University of Cologne Cologne Germany; ^21^ MGH Neurosurgery and Center for Neurotechnology and Neurorecovery (CNTR) at MGH Neurology Massachusetts General Hospital Harvard Medical School Boston MA USA

## Abstract

**Objective:**

Accurate electrode placement and individual stimulation parameters influence the outcomes of subthalamic deep brain stimulation in Parkinson's disease. Neuroimaging‐based models can help evaluate how electrode placement impacts improvement, aiming to reduce the burden of programming. However, most existing models have been developed to explain differences between patients rather than differences between contacts within the same patient, leaving the clinical relevance of image‐guided programming unclear.

**Methods:**

We analyzed data from patients with Parkinson's disease treated with subthalamic deep brain stimulation to develop and validate a neuroimaging‐informed model of motor improvement measured by the Unified Parkinson's Disease Scale. Five approaches were tested: active contact coordinates, electric fields, tract activations, as well as structural and functional networks. All approaches were integrated into a combined ridge regression model and validated using 2 hold‐out datasets.

**Results:**

The sample included 236 patients (604 stimulation sites), divided into a training cohort (N = 129), a retrospective validation cohort (N = 89), and a prospectively acquired validation cohort (N = 21 electrodes). Consistent with expectations, our model explained approximately 12% of the variance in unseen group‐level data (*R* ^2^ = 0.12, *p* = 0.001). At the individual level, the model identified the optimal clinical contact or its neighboring contact in all but one case (mixed‐effects *R* ^2^ = 0.31, *p* = 3.67 × 10^−10^).

**Interpretation:**

An imaging‐informed model explained the expected variance at the group level and demonstrated potential for guiding stimulation programming, suggesting that image‐guided approaches may improve clinical decision making while reducing the need for lengthy postoperative testing. ANN NEUROL 2026;100:22–35

Deep brain stimulation (DBS) is an effective treatment option for Parkinson's disease (PD), but achieving the best outcomes requires precise electrode placement and carefully selected stimulation parameters.^1^ DBS programming identifies the electrode contact or combination of contacts that provide the greatest therapeutic effect. In most centers, this process is time‐consuming and burdensome, often requiring multiple follow‐up visits, especially during the first year after surgery.^2^ Several strategies have been proposed to streamline this process, including electrophysiological sensing^3^, ^4^ and image‐guided programming.^5^, ^6^ Image guidance maps the electrode's location relative to brain anatomy,^7^ and may be informed by probabilistic “sweet spots,”^8^ optimal sets of structural connections,^9^, ^10^ or polysynaptic whole‐brain network targets.^11^ First, randomized controlled clinical trials have shown that image‐guided programming can achieve noninferior results compared to expert‐based DBS programming.^6^

The performance of image‐guided models has often been evaluated by their ability to explain variance in clinical outcomes across unseen group‐level cohorts.^9^, ^11^, ^12^, ^13^ Whereas this provides insight into DBS mechanisms and helps explain some variance in clinical outcomes, applying such models in a clinical setting requires accurate predictions on the individual level, posing fundamentally different challenges. In clinical practice, these models can be applied to suggest suitable electrode contacts or stimulation parameters to optimize care in individual patients. Consequently, some studies have already quantified how well algorithm‐recommended contacts align with those selected by expert clinicians. Importantly, the ultimate aim in this case is to match expert‐based decisions or even to improve outcomes in individual patients.^5^, ^6^, ^9^ Although they are related, these 2 tasks represent distinct goals. First, explaining group‐level variance and, second, improving individual patient outcomes each present different challenges to image‐guidance models.

The first task (explaining group‐level outcomes) is complicated by the many factors that influence DBS outcomes beyond active contact placement (assuming the electrode reaches the target nucleus). In our focused literature review, we identified numerous sources of heterogeneity in clinical outcomes after DBS, including disease subtypes, duration, structural and anatomic variations, comorbidities, and more. We must emphasize that these considerations are specific to subthalamic nucleus DBS (STN‐DBS) for PD, and may largely differ for other indications.^14^, ^15^ Here, group‐level data often suffer from inter‐ and intra‐rater variability in clinical scoring and suboptimal clinical imaging, especially when aggregated across multiple centers (Fig 1A). Whereas some studies compared the impact of several factors, no single study exhaustively quantified their relative contributions in a combined analysis. This introduces an unclear covariance structure, which requires assumptions about factors such as disease subtype or imaging quality (eg, due to head motion). These assumptions introduce uncertainty and potential bias. Although it remains unclear how much of the variance in clinical response image‐guidance models can explain in heterogeneous (“noisy”) group‐level data, based on our literature review, we hypothesize that approximately 10% of the variance in clinical outcomes in such data can be attributed to electrode placement.^9^ A more detailed discussion of this analysis may be found in the Supplementary Text S1 and Supplementary Table S1.

**Figure 1:**
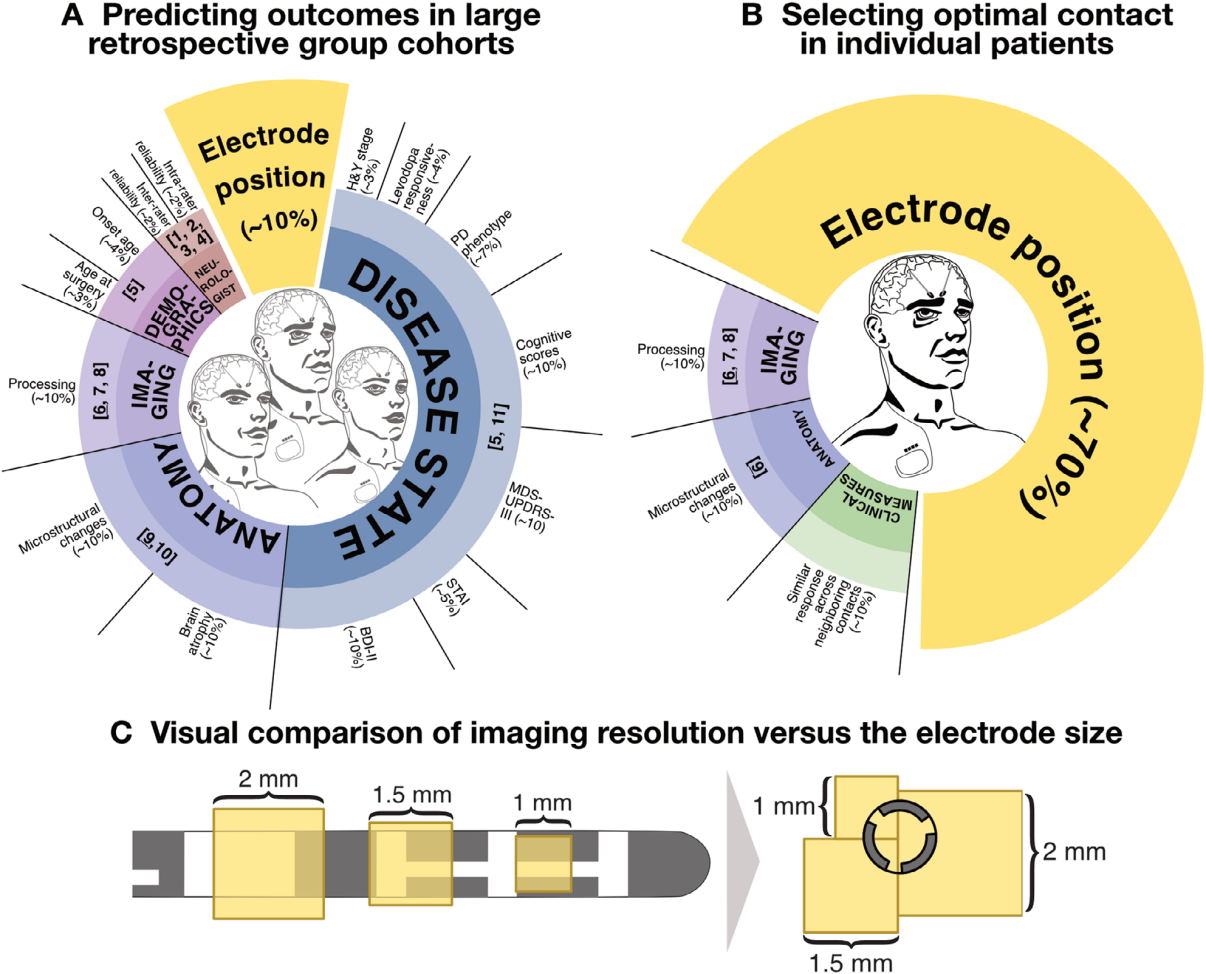
Factors influencing long‐term clinical outcomes and accuracy of STN‐DBS image guidance models. (A) Our focused literature search suggested that we lack conclusive evidence on how various factors complicate the task of estimating clinical improvements across heterogeneous group‐level STN‐DBS datasets in the treatment of Parkinson's disease (PD). The identified contributing factors include disease state, macro‐ and microscopical anatomical brain abnormalities, imaging*, demographics, and examiner/neurologist. Based on estimates from the literature, we expected the electrode position to explain approximately 10% of the variance in clinical improvements (considering it reaches the defined target nucleus). The numbers in the figure were approximated, based on the factors deemed influential in the literature (see Supplementary Text S1 and Supplementary Table S1 for source details). The innermost circle includes the specific category, the middle circle includes the reference number (see Supplementary Table S1), the outermost circle includes the specific factor. (B) When DBS imaging guidance tools are tasked with suggesting optimal DBS contacts in individual patients, the structure of nuisance variables differs significantly. Numerous noisy/heterogeneous factors populating group‐level data (A) are eliminated, because the model must select options within the same patient, same brain, and same subthalamic nucleus. However, we estimated that image guidance models could still be affected by microstructural anatomical changes. For instance, perivascular (Virchow‐Robin) spaces located closer to some of the contacts would lead to changes in outcomes that the guidance model cannot predict. Furthermore, the task is nontrivial to begin with, since neighboring electrode contacts will often lead to comparable clinical responses, i.e., there may not always be an unequivocal “top contact”, leading to some degree of ambiguity in the data. We therefore attributed an additional 10% of the variance to such clinical measurement uncertainty. Lastly, variance arising from imaging data processing remains consistent across both group‐level and individual‐level modeling scenarios. Therefore, while optimal contact selection is not the sole contributor to clinical improvement, we hypothesize it explains a substantial 70% of the variance in clinical improvement in a single patient. All references can be found in the Supplementary material (Supplementary Text S1 and Supplementary Table S1). (C) Whether explaining variance in clinical improvement at the group level, or suggesting optimal stimulation contacts in an individual patient, imaging resolution remains a key limiting factor, especially when making choices across individual contacts on a single electrode. The required resolution may theoretically exceed that of imaging currently used in clinical practice. For example, resolution in typical structural MRI is around 1 mm^3^, in diffusion MRI between 1–2 mm^3^ and in functional MRI between 2–3 mm^3^. The spacing between the centers of adjacent contacts on a typical DBS electrode is about 2 mm, with segmented contacts being placed even closer together. As a result, a single voxel may often cover multiple DBS contacts, highlighting the challenges imaging resolution poses for the accuracy of image‐guided DBS programming. H&Y stage = Hoehn and Yahr Scale; MDS‐UPDRS‐III = MDS Unified Parkinson's Disease Rating Scale; STAI = State Trait Anxiety Inventory; BDI‐II = Beck Depression Inventory. *It is important to note that no study has deliberately tested for imaging processing or reconstruction errors. However, multiple reports speak of reconstruction errors displacements of at least 1 mm. To our knowledge, the only study that tested for artificially introduced spatial uncertainty imposed a jitter of *N* = 258 electrode placements using a 3D Gaussian distribution with a 2 mm full width half maximum. Models created after spatial jittering correlated with the original model at approximately *R* = 0.8, corresponding to 36% non‐shared variance between the original and jittered models. However, only a fraction of this unexplained variance can be attributed to neuroimaging‐related spatial uncertainty, as additional factors such as random noise or imperfect model fitting are also expected to contribute. Therefore, we estimate that neuroimaging‐related uncertainty accounts for approximately 9% of the variance in clinical improvement following DBS. [Color figure can be viewed at www.annalsofneurology.org]

The second task (selecting optimal stimulation parameters in an individual patient) eliminates many of the confounding variables that populate the first task. For instance, patient‐specific characteristics, such as PD subtype, disease duration, and age, stay constant for any given patient. Additionally, the same clinician may assess responses to stimulation at each contact within a single session, eliminating observer bias (Fig 1B). However, this individualized approach introduces novel challenges that are different in nature. Namely, contacts along the same electrode are merely millimeters apart from one another, and neighboring contacts will often produce comparable stimulation results. Therefore, identifying a single best contact may be limited by imaging resolution, especially when segmented electrodes are used (Fig 1C). Furthermore, microstructural anatomic variations unknown to the model, such as Virchow‐Robin spaces, may still cause unexpected clinical outcomes, even with optimal electrode placement and contact selection.^16^ Nonetheless, if image‐guided models are to be used to inform DBS programming, these challenges must be overcome. Based on these considerations (see Supplementary Text S1 for details), we hypothesized that image guidance models would have clear clinical value in informing programming decisions for individual patients.

To empirically test these theoretical considerations, we developed a multimodal image‐guided model based on a large multicenter cohort and evaluated its performance in the 2 aforementioned tasks: (1) estimating and explaining variance in clinical outcomes in an independent group‐level cohort, and (2) suggesting optimal stimulation contacts for individual patients. After constructing the model, our first aim involved testing it on between‐subject variance. Our second aim sought to improve clinical efficiency, as identifying a subset of optimal clinical contact(s) could significantly reduce DBS programming time.^6^ Within this effort, we constructed and validated the image‐guidance model informed by 5 distinct methods of analysis, namely anatomic coordinates, “sweet spots,” optimal tract connections, and distributed structural and functional networks, using data from 236 patients (604 stimulation sites) across 5 international DBS centers.

## Materials and Methods

### Patient Cohorts, Imaging, and Clinical Assessment

We collected an overall sample of 247 patients with bilateral STN‐DBS treated for PD from 6 different cohorts. Of these, data from 236 patients were retained for the analyses (Supplementary Fig S1). Informed consent was obtained in accordance with the Declaration of Helsinki, and the study methods have been approved by the Institutional Review Board at Charité‐Universitätsmedizin Berlin. The discovery dataset consisted of 129 retrospectively enrolled patients from 3 centers. The first validation dataset included 2 additional retrospective cohorts comprising 89 patients. The second validation dataset consisted of 18 patients in whom the clinical response of each contact was tested at 2 mA (N = 21 electrodes) as part of clinical trial (DRKS00026596) at the University Hospital Cologne.

Clinical symptoms (off medication) before and after surgery were assessed using the total motor score of the Unified Parkinson's Disease Rating Scale (UPDRS III) for all patients, except the contact‐wise stimulation cohort, in which upper limb contralateral hemi‐scores were acquired based on the rigidity, finger tapping, resting tremor, postural tremor, and kinetic tremor items of the Movement Disorder Society (MDS)‐UPDRS III, rated by a clinician who was blinded to the current stimulation settings. The contact‐wise stimulation cohort was derived during the routine monopolar review process. A full MDS‐UPDRS III assessment was impractical due to time constraints. The unilateral focus of this cohort also reflects the decision to limit testing to the hemisphere contralateral to the tremor‐affected upper extremity.

### DBS Electrode Localization and Electric Field Calculation

DBS electrode location was reconstructed using Lead‐DBS version 3.1 software run in MATLAB R2022b (The MathWorks Inc., Natick, MA), following an advanced, state‐of‐the‐art protocol.^1^ Pre‐ and post‐operative images were first linearly co‐registered using Advanced Normalization Tools (ANTs)^17^ followed by nonlinear normalization into the ICBM 2009b Nonlinear Asymmetric (“MNI”) template space using ANTs SyN,^18^ and SPM, as implemented in Lead‐DBS.^1^ Normalization misalignments were corrected using WarpDrive.^19^ Potential intraoperative brain shift was corrected using subcortical refinements of the registration.^20^ In the case of postoperative computed tomography (CT; N = 201) images, the electrode placements were reconstructed using the phantom‐validated Precise and Convenient Electrode Reconstruction for Deep Brain Stimulation (PaCER) approach.^21^ Electrodes from postoperative magnetic resonance imaging (MRI) scans (N = 46) were reconstructed using the TRAC/CORE algorithm. If necessary, results from both methods were manually inspected and refined.^22^ The DiODe algorithm^23^ was used to estimate the rotational orientation of directional leads, followed by manual inspection and adjustment when needed.

Stimulation volumes were approximated by calculating electric field magnitudes (E‐fields) using Open Source Simulation (OSS)‐DBS version 2.^24^ E‐field calculation approximates voltage gradients administrated from the electrode to voxels in space, yielding higher values closer to its source and lower values with increased distance from the source. OSS‐DBS was used via its integration into Lead‐DBS, and the modeling details can be found in Butenko et al.^24^ Stimulation volumes were estimated in native space and warped to template space using the same nonlinear registration field described earlier.

### Statistical Analysis

#### Models Associated With the Optimal Clinical Improvement

Models associating stimulation sites with clinical improvements were calculated based on the discovery cohort (N = 129) using 5 methods of increasing modeling complexity. First, at the electrode modality, active contact locations were averaged per hemisphere and weighted by their improvement. This yielded a single “optimal” coordinate. Second, at the stimulation volume modality, we correlated patient‐specific E‐fields with clinical improvements using established DBS Sweet Spot Mapping.^13^ For the third to fifth modalities, we applied connectomic approaches implemented in established methods called DBS Fiber Filtering^25^ and DBS Network Mapping,^11^ to identify optimal streamlines, structural network, and functional network targets. All details are provided in the Supplementary Methods S1.

#### Validation of the Models and Calculation of Surrogate Values

Model performance at all modalities was first assessed in a circular (in‐sample) manner, then evaluated using 10‐fold cross‐validation (CV) within the discovery dataset. Models for each modality (coordinates, E‐fields, tracts, and structural/functional networks) generated estimates that were optimized to be strongly correlated with the real clinical improvement for each patient. These estimated values did not directly predict UPDRS III improvement but rather represented surrogate variables (or scores) that would optimally be correlated with UPDRS III improvement values. For example, on the coordinate modality, the surrogate for the clinical scores was expressed by the proximity of the patient specific chronic contact to the optimal coordinate. The closer the stimulation site to the target, the better the outcome would be, according to the model. For the remaining methods, surrogate variables represented Pearson's correlations between the model and patient profile (for more details see Supplementary Methods S2). Because these surrogate values were calculated from the entire discovery dataset, this was a circular (in‐sample) analysis. Next, the procedure was repeated using a 10‐fold CV design, applied analogously at the coordinate, E‐field, tract, and network modalities. Using the coordinate modality as an example, the discovery dataset was split into 10 folds. The optimal stimulation coordinates were iteratively calculated based on 9 folds, and proximity to the resulting coordinate was measured in the left‐out 10th fold. This procedure was repeated for each left‐out fold. This ensured the data from the estimated (hold‐out) fold were not included in the optimal stimulation target calculation, preventing circularity. This CV was carried out across all modalities, strictly taking care that no data leakage was possible in any of the processing steps. Note that we deliberately refrain from providing *p* values for in‐sample analyses throughout the manuscript, but we provide *p* values when models were subjected to CV (circumventing circularity).

#### Combined Ridge Regression Model of Clinical Improvement

All 5 models relied on DBS electrode localization, which can lead to high covariance across the model estimates. However, prior research suggests that combining different modeling strategies may still explain additional outcome variance.^11^ Each method likely has unique pitfalls that can lead to false predictions in individual cases. We hypothesized that combining all 5 strategies into a joint model could enhance robustness and better generalize to unseen data by reducing the risk of overfitting to method‐specific noise. To do so, we fit ridge regression (using a regularization parameter of *λ* = 50, see Supplementary Methods S3 for more details) to the full training dataset using the in‐sample surrogate values from all 5 models. Ridge regression was chosen to address multicollinearity, reduce overfitting, and increase predictive power on the out‐of‐sample validation dataset. Multicollinearity of our scores arose not only from overlapping anatomic or functional information across different modalities, but also from shared variance introduced by our preprocessing pipelines.

#### Validation of the Combined Model Using an Independent Dataset

Each of the 5 models was tested on a previously unseen, heterogeneous (hold‐out) test dataset from 2 different centers and a prospectively acquired contact‐wise stimulation cohort. Model‐derived surrogate variables for clinical improvement in the test cohort were generated using models trained exclusively on the discovery dataset. Specifically, Euclidean distance and Pearson's correlation coefficients were calculated in the same way as in the discovery cohort. These model‐derived surrogates were then used to (1) test the performance of each model individually across the 5 modalities, and (2) test the combined performance using the regression model.

First, the ridge regression model, trained on the normalized surrogate variables of the discovery cohort, was used to predict clinical outcomes in the retrospective validation dataset. Importantly, this was done without refitting the model to the validation dataset in any way; that is, the *ß* coefficients were calculated exclusively based on the discovery cohort.

The combined ridge regression model was also evaluated using prospectively acquired patients who were stimulated using a fixed amplitude of 2 mA on each of the 8 contacts. Here, each electrode had 8 clinical outcome values (one for each contact) and was treated as an individual dataset. As in the retrospective validation cohort, each contact received 5 model‐derived surrogate values (proximity to the optimal coordinate and various Pearson's correlation coefficients with the sweet spot, optimal streamline profile, and structural and functional network profiles). These 5 surrogates were then used to derive clinical improvement in the test cohort, based on the beta estimates from the ridge regression model trained exclusively on the discovery cohort.

To evaluate the relationship between estimated and empirical clinical improvements in the prospectively acquired contact‐wise stimulation cohort, we fitted a linear mixed‐effects (LME) model using MATLAB's *fitlme* function. This analysis was performed in this cohort, as each electrode contributed 8 stimulation sites per patient, allowing repeated measures to be modeled within subjects. The model formula was defined as:

(1) $$\text{Empirical}\;{\text{Improvement}}_{\mathrm{ij}}=\beta_{0}+\beta_{1}\cdot \text{Estimated}\;{\text{Improvement}}_{\mathrm{ij}}+\mu_{0j}+\varepsilon_{\mathrm{ij}}$$

where *i* represents contacts and *j* represents electrodes. Empirical improvement represents the dependent variable (clinician‐rated improvement per contact). ß_*0*_ represents the overall baseline across all electrodes, whereas ß_*1*_ × Estimated Improvement *ij* represents the systematic linear relationship between the model's predictions and empirical data. Estimated Improvement is the fixed‐effect predictor derived from the ridge‐regression model. The terms μ_*0j*_ and ε_*ij*_ correspond to the per‐electrode random intercept (accounting for between‐electrode variability) and residual (within‐electrode) error, respectively.

The estimated improvement was modeled as a fixed effect, and electrode identity (Lead) was modeled as a random intercept (1|Lead) to account for within‐patient dependence. The intercept term represents the baseline empirical improvement for each electrode when the predicted improvement equals zero. All variables were z‐scored prior to model fitting.

As a sensitivity analysis, we also fitted a linear mixed‐effects model including random intercepts for patients and electrodes nested within patients *(1|Patient) + (1|Patient:Lead)*.

To assess predictive performance,^26^ we calculated the coefficient of determination (*R* ^*2*^) as *R* ^*2*^ = 1 − Residual sum of squares/total sum of squares in the first out‐of‐sample validation cohort.

(2) $$R^{2}=1-\frac{\sum {\left(y_{i}-\hat{y_{i}}\right)}^{2}}{\sum {\left(y_{i}-\bar{y}\right)}^{2}}$$

Where $\sum {\left(y_{i}-\hat{y_{i}}\right)}^{2}$ is the residual sum of squares, representing the squared difference between empirical and predicted values, and $\sum {\left(y_{i}-\bar{y}\right)}^{2}$ is the total sum of squares, representing the variance of the empirical values around their mean.

## Results

### Patient Demographics and Clinical Results

The discovery dataset included 129 patients with PD with STN‐DBS from 3 cohorts (Berlin = 51, Würzburg = 44, and Amsterdam = 34). The retrospective, heterogeneous group‐level validation dataset included 2 cohorts (Beijing = 41 and Würzburg = 48). The second validation cohort included 18 prospectively acquired contact‐wise stimulation data (21 electrodes). Clinical scores, demographics, and active contact placements are provided in Supplementary Tables S3, and S4, and Supplementary Figure S5.

### Optimal DBS Target Sites Across Multiple Modalities

**Figure 2:**
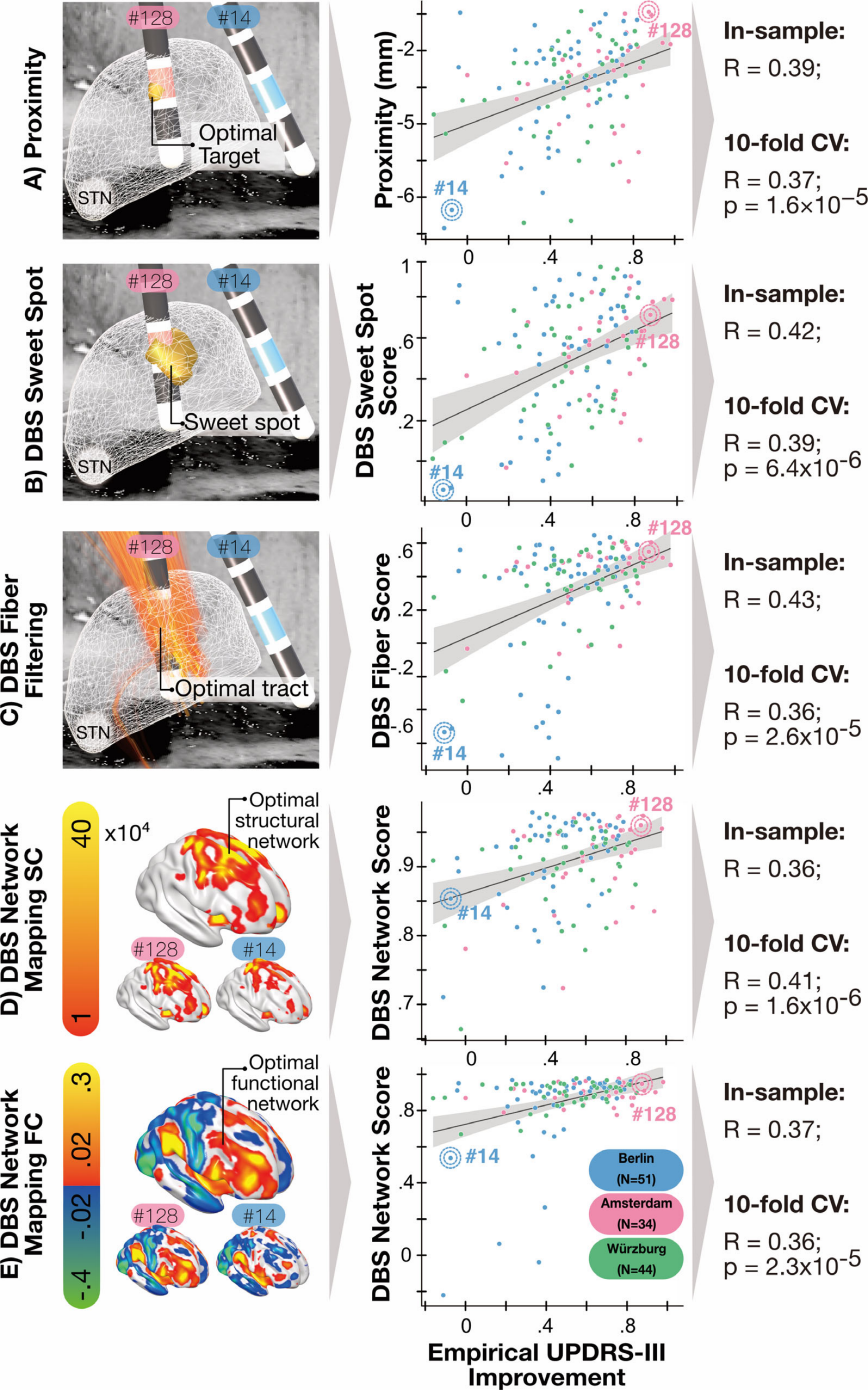
Imaging‐based DBS models and their association with clinical outcomes. Five distinct methods were used to generate models that associated clinical improvements with the stimulation site. The validity of these models was examined in a circular manner (in‐sample) and via 10‐fold cross‐validation (CV). *P*‐values are not reported for in‐sample calculations due to the circular nature of these results. The left column of the figure depicts a 3D visualization of the model showing regions associated with the clinical improvement. The positions of electrodes with labeled stimulated contacts (A–C) along with network profiles (D, E) are shown for two example patients (patient #14 with low empirical improvement and #128 with high empirical improvement). The middle column shows the correlation between the model‐based scores and the actual empirical improvements. The positions of the two example patients in the correlation plots are indicated by circles. The grey shaded area in the plot represents a 95% confidence interval. (A) Patients with electrodes closer to the optimal stimulation coordinate (Proximity), as calculated by the Euclidean distance, had higher relative clinical improvement. (B) Patients whose E‐fields peaked in regions overlapping with the sweet spot received higher scores (DBS Sweet Spot Scores), while those with E‐fields that peaked elsewhere tended to receive lower scores. (C) Patients whose streamline profile correlated with the modeled optimal streamline profile (DBS Fiber Score) showed on average higher empirical clinical improvements. (D) Structural network maps associated with clinical improvement showed stronger correlations with connectivity profiles (DBS Network Score) in patients with higher empirical clinical improvements. (E) Optimal functional connectivity profiles more closely resembled the DBS‐related patient network profiles (DBS Network Score) in patients with higher empirical clinical improvement. STN = subthalamic nucleus, CV = cross‐validation, SC = structural connectivity, FC = functional connectivity, DBS = deep brain stimulation. [Color figure can be viewed at www.annalsofneurology.org]

Optimal target engagement was measured at the coordinate, stimulation volume, tract, and structural and functional network modalities in the discovery dataset. As expected, the optimal coordinate and the sweet spot were found in the dorsolateral motor STN. Optimal structural connectivity profiles peaked in the supplementary motor area (SMA), pre‐supplementary motor area (pre‐SMA), and superior frontal gyrus, whereas functional connectivity profiles were anticorrelated with the primary motor cortex (M1) (Supplementary Results S1 and Supplementary Fig S6). At the coordinate modality, the proximity of each patient's active contact significantly correlated with clinical improvements (in‐sample: *R* = 0.39, 10‐fold CV: *R* = 0.37, *p* = 1.6 × 10^−5^). This indicates that the closer the active contact to the optimal stimulation coordinate, the higher the improvement (Fig 2A). At the sweet spot modality, the spatial correlation between each patient's E‐field and the optimal sweet spot profile also led to significant results (in‐sample: *R* = 0.42, 10‐fold CV: *R* = 0.39, *p* = 6.0 × 10^−6^; Fig 2B). When repeating sweet spot mapping without mirroring stimulation volumes across hemispheres, peak stimulation regions are largely unchanged and CVs remain significant (in‐sample *R* = 0.48, 10‐fold CV *R* = 0.33, *p* = 5.9 × 10^−4^; out‐of‐sample *R* = 0.24, *p* = 0.029). At the tract modality, patients whose stimulation overlapped with the optimal tract profiles had better clinical outcomes (in sample: *R* = 0.43, 10‐fold CV: *R* = 0.36, *p =* 2.9 × 10^−5^; Fig 2C). For DBS Sweet Spot and DBS Fiber Filtering analyses, testing different thresholding options led to comparable results (Supplementary Results S1, Supplementary Figs S7 and S8). At the network modality, stimulation profiles that were more closely correlated with the identified structural (structural in‐sample: *R* = 0.36, 10‐fold CV: *R* = 0.41, *p =* 1.6 × 10^−6^) and functional networks (functional in‐sample: *R* = 0.37, 10‐fold CV: *R* = 0.36, *p* = 2.3 × 10^−5^) yielded higher clinical outcomes (Fig 2D, E). Overall, the similarity between in‐sample and CV *R*‐values suggests a minimal degree of overfitting in any of these models.

### Predicting Outcomes in Unseen Heterogeneous Group‐Level Data

**Figure 3:**
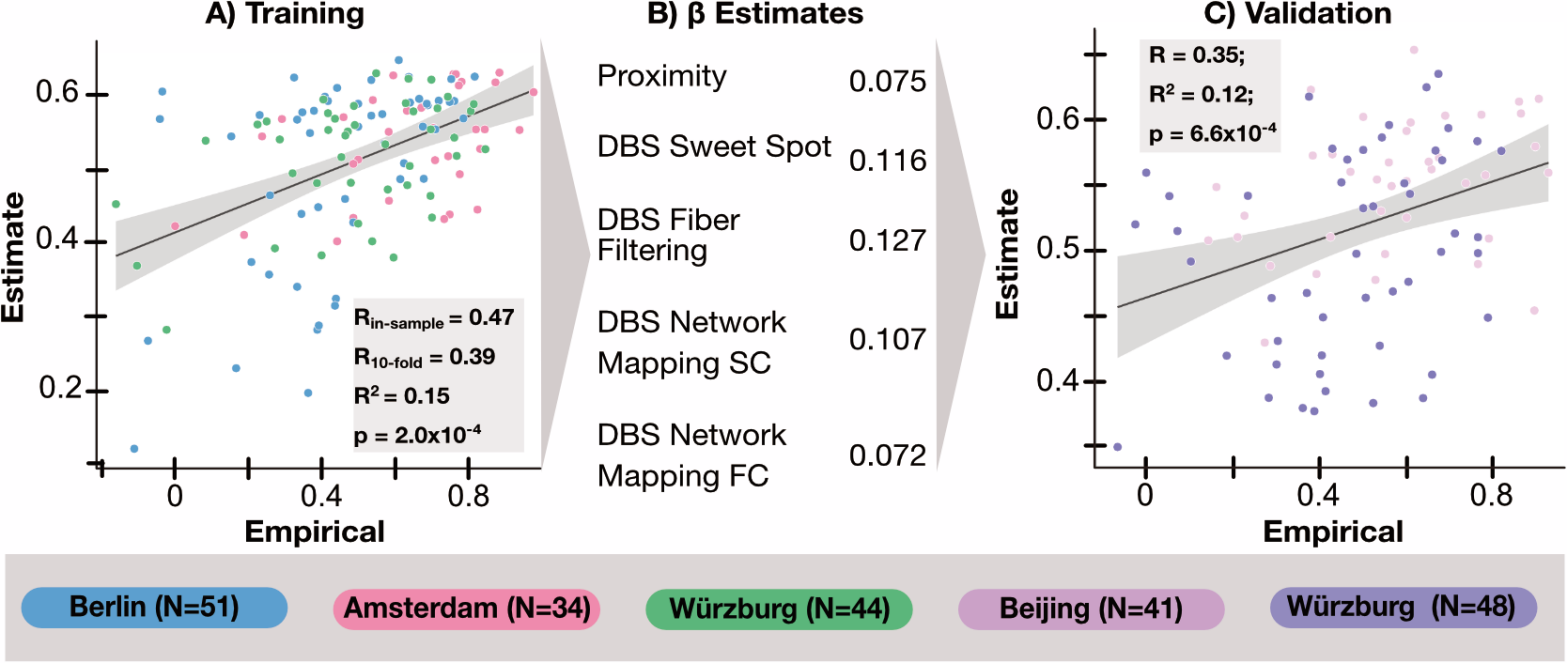
Validation of a combined model of optimal deep brain stimulation (DBS) profile. Our combined model, trained on the discovery dataset, was used to estimate clinical improvements in unseen patients. The model was fitted using the ridge regression method with a *λ* parameter of 50 (Supplementary Methods S2, Supplementary Figure S2). The grey shaded area in the plot represents a 95% confidence interval. (A) The optimal stimulation models, determined by a data‐driven method, significantly correlated with the empirical clinical improvements both in in‐sample and in a ten‐fold cross‐validation design. (B) The strongest predictor of clinical improvement was the fiber filtering score (*ß* = 0.127), followed by sweet spot score (*ß* = 0.116), structural connectivity network mapping score (*ß* = 0.107), proximity to an optimal coordinate (*ß* = 0.075), and functional connectivity network mapping score (*ß* = 0.072). (C) This model's estimates explained 12% of the variance in an out‐of‐sample validation. [Color figure can be viewed at www.annalsofneurology.org]

The joint model explained 22% of variance in clinical improvement within the discovery cohort (in‐sample: *R* = 0.47, *R* ^*2*^ = 0.22) and 15% using 10‐fold CV (*R* = 0.39, *R* ^*2*^ = 0.15, *p* = 2.0 × 10^−4^; Fig 3A). The predictors influenced the clinical scores in the following order: DBS Fiber Filtering score, DBS Sweet Spot score, DBS Network Mapping SC score, Proximity, and DBS Network Mapping FC score (Fig 3B, stability of *ß* estimates is shown in Supplementary Methods S3 and Supplementary Fig S4). In the validation dataset, the model explained 12% of variance in clinical improvement (*R* = 0.35, *R* ^*2*^ = 0.12, *p* = 6.6 × 10^−4^; Fig 3C). See results for individual methods in the Supplementary Results S2. Importantly, the variance was not obtained by squaring the *R*‐value, but by computing the coefficient of determination, in line with the community standards proposed to demonstrate evidence of “prediction.”^26^ Alternatively, squaring the *R*‐value yielded  an *R* ^2^ value that was identical when rounded to two decimals (*R* = 0.35: *R* ^*2*^ = 0.12 at *p* = 6.6 × 10^−4^). This value could be interpreted as the amount of variance explained in ranks of outcomes across the unseen cohort. Whereas all modalities contributed to the ridge regression, fiber filtering and sweet spot mapping had the strongest impact on the model performance.

### Matching Optimal Electrode Contacts in Prospectively Acquired Patients

**Figure 4:**
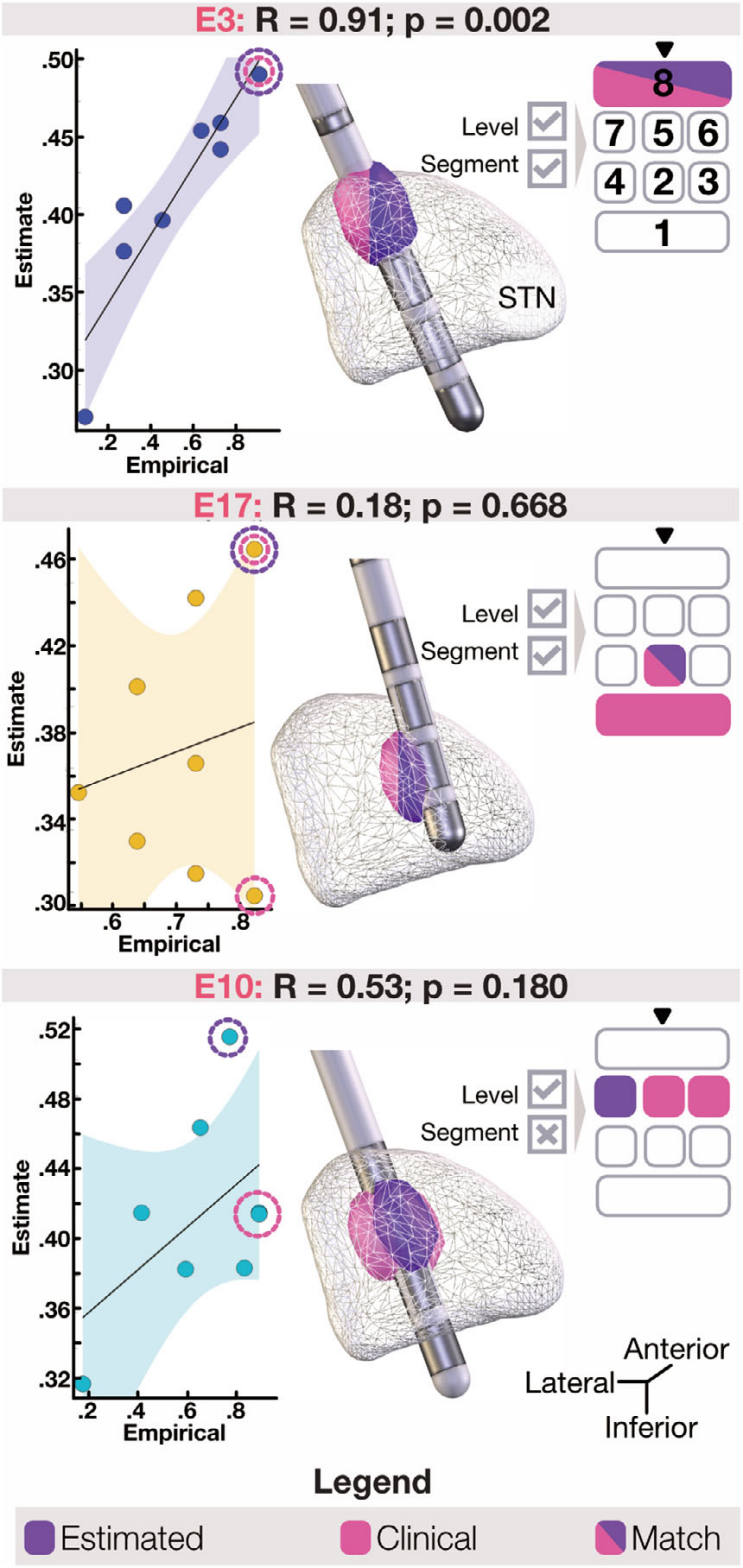
Estimates of clinical improvement in patients with Parkinson's disease in the contact‐wise stimulation cohort. The combined model was used to estimate clinical improvements for 21 electrodes based on contact‐wise stimulation data. Each contact had been assigned an empirical clinical improvement by the clinician. Three example patients are shown. For each patient, we show correlation plots between the model‐estimated clinical improvement for each contact on the electrode and the empirical clinical improvement assigned by the neurologist (left side of the figure). In the center, we show the electrode's position within the subthalamic nucleus (STN; white mesh) and stimulation volumes from the model‐selected and empirically selected optimal contacts. A schematic visualization is shown on the right side. Downward‐facing black triangles indicate the location of the electrode marker. In two of the three patients, clinicians assigned identical empirical clinical improvement scores to two contacts. Scores were still assigned individually to each contact during the monopolar review process. For patients E3 and E17, the estimated clinical contact matched the empirical one. For patient E10, the estimated contact did not match the empirically selected one but matched the level at which the contact was located. [Color figure can be viewed at www.annalsofneurology.org]

**Figure 5:**
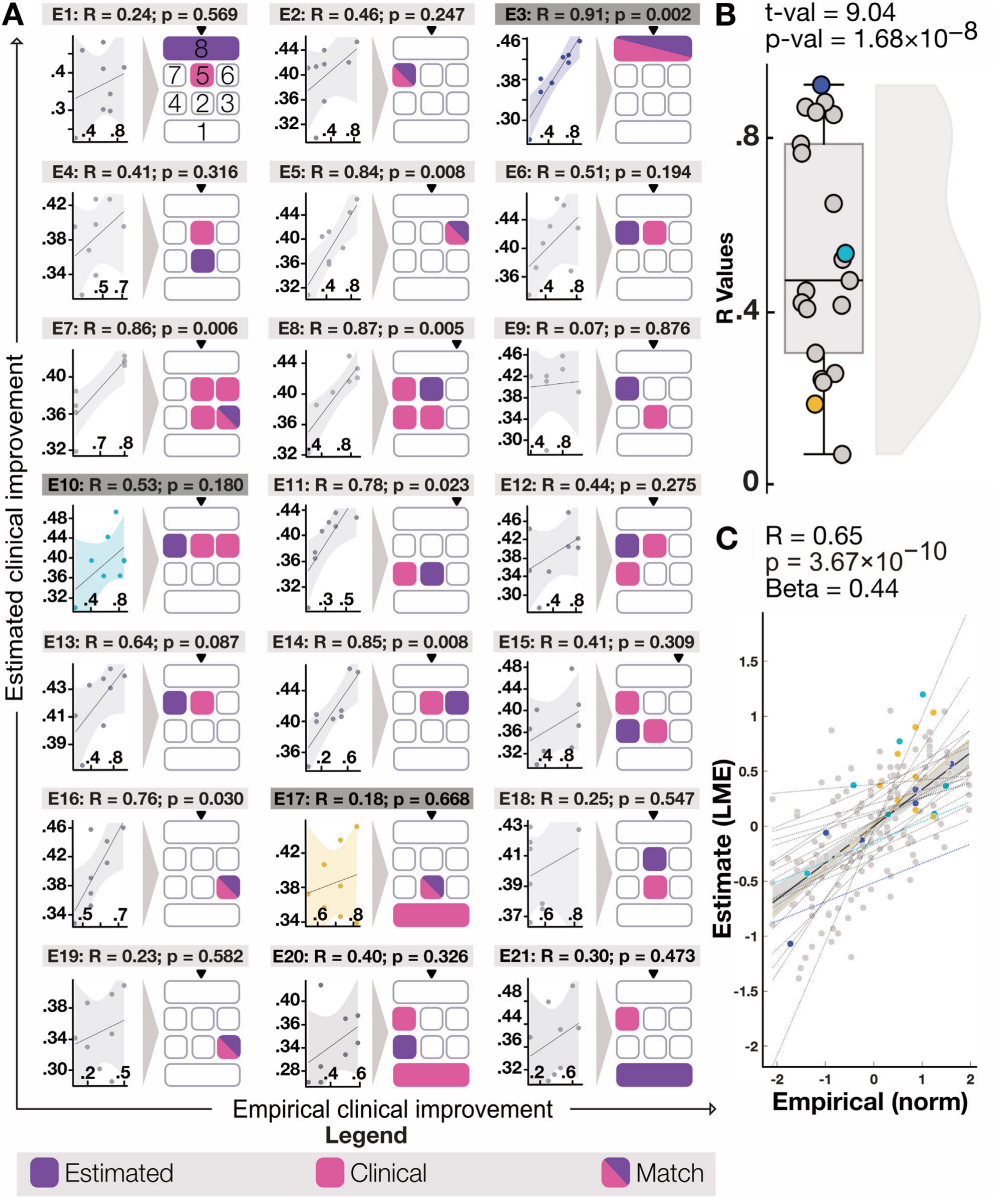
Estimates of clinical improvement for each electrode in the contact‐wise stimulation cohort. The combined model estimated clinical improvements for individual contacts of 21 electrodes (labeled as E1–E21) based on their specific placements. (A) In the correlation plots, each point represents an electrode contact, with the x‐axis showing its empirical clinical improvement and the y‐axis displaying the estimated improvement. The highlighted plots (E3, E10, E17) correspond to the electrodes shown in more detail in Figure 4. To the right of the correlation plots, each electrode is shown with its eight contacts (1 to 8), indicating whether it was identified as the contact with the highest clinical improvement. Downward‐facing black triangles indicate the location of the electrode marker. (B) Distribution of correlation values between model estimates and empirical clinical improvements. (C) Results of a linear mixed‐effects model, with intercept adjusted for each electrode. [Color figure can be viewed at www.annalsofneurology.org]

The model explained 12% of variance in the retrospective, heterogeneous (“noisy”) hold‐out test cohort, matching our literature‐informed theoretical expectations as well as the approximate explained variance in smaller studies with comparable aims.^1^, ^9^, ^10^, ^13^, ^27^ However, the key question of whether our model could effectively inform DBS programming in individual patients remained open. To test this, we analyzed a prospectively collected dataset in which each of 8 contacts on 21 segmented DBS electrodes (Boston Scientific Vercise Cartesia) was stimulated at a fixed amplitude of 2 mA. Clinical responses were recorded after overnight withdrawal of dopaminergic medication.^28^, ^29^ Contralateral clinical responses for each electrode were first analyzed separately by (i) correlating the model estimates with clinical improvements across the 8 contacts and (ii) comparing the contact with the highest estimated score with at least one of the best clinical contacts (Figs 4 and 5A). The average correlation value across electrodes was 0.52 ± 0.26 (range = 0.07–0.91; Fig 5B). All correlations were positive. High correlations (*R* > 0.70) were present in 7 cases. Whereas, in some cases, correlations were weaker (eg, *R* = 0.18, *p* = 0.668; see Fig 4; E17), the model still correctly identified the optimal clinical contact. Conversely, in other instances, a comparatively high correlation of *R* = 0.53, *p* = 0.180 (see Fig 4; E10) did not correctly identify the optimal contact. We must emphasize that these data involved directional leads, where a stimulation level refers to the ring of the lead. Identifying the optimal stimulation level would already prove clinically relevant by reducing programming time.^6^ Our model achieved this success in 71% of cases (chance level 32%, *p*_binomial_ = 0.0003). The optimal contact was correctly selected as the top one 33% of the time (chance level = 18%, *p*_binomial_ = 0.0278), selected in the top 2 contacts 67% of the time (chance level = 35%, *p*_binomial_ = 0.0281), and in the top 3 contacts 86% of the time (chance level = 48%, *p*_binomial_ = 0.0025). Finally, contacts associated with the lowest empirical improvement were ranked lower than those with highest empirical improvement in 90% of cases. Figure 5A shows details of estimated and empirical contacts for all cases. Results of individual methods are in the Supplementary Results S2, Supplementary Figure S9, and Supplementary Table S6. As can be visually appreciated, the model suggestions were neighboring or matching the empirical suggestions in all but one electrode. In this electrode (E21), the suggested contact was not adjacent to the best clinical contact but was itself the second‐best clinical contact.

To more formally quantify the correspondence between model‐estimated empirical improvements across all electrodes, we fitted an LME model treating the predicted improvement as a fixed effect and electrode identity as a random intercept to account for within‐patient dependence. The LME with the electrode as a random intercept revealed a strong positive relationship between estimated and empirical improvements (*R* = 0.65, *R* ^*2*^ = 0.31, *p* = 3.67 × 10^−10^). The standardized slope (*ß* = 0.44) indicated that higher model estimates were associated with higher empirical improvements across contacts. When repeating the LME analysis after adding “patient” as an additional random intercept (because 3 patients had contributed 2 electrodes into the analysis), results were comparable (*R* = 0.63, *R* ^*2*^ = 0.32, *p* = 3.78 × 10^−10^). However, the slope being less than 1 shows that the model tended to underestimate the absolute magnitude of improvement differences (the predicted values varied over a narrower range than the empirical scores), whereas still accurately capturing their direction and relative ranking (Fig 5C). The model was also able to capture variance in both akinesia and tremor symptoms separately (Supplementary Fig S10).

## Discussion

Image guidance is a promising approach to maximize the efficacy of DBS. However, the exact optimal strategy for guiding DBS programming remains unclear, with numerous approaches proposed in the scientific literature.^5^, ^9^, ^30^ Although the concept seems like a natural choice, good evidence for clear clinical utility is lacking.^31^ To address this gap, we leveraged multiple state‐of‐the‐art concepts to analyze the effects of an exceptionally large cohort of 604 subthalamic stimulation sites to create and validate DBS models associated with clinical benefit in PD. Both prior literature and the present empirical results suggested that image guidance models could be expected to account for approximately 10% of variance in clinical improvements in an unseen heterogeneous group‐level retrospective STN‐DBS cohort of patients with PD. Importantly, this held true even for our advanced multi‐scale model, which combined imaging predictors from 5 neuroimaging modalities using highly sophisticated methods. However, when controlling for numerous nuisance factors (sources of “noise”) present in typical group‐level data, the same model was able to suggest correct or neighboring DBS contacts in all but one examined individual patients.

These results suggest clear relevance of image guidance for clinical practice when used to identify optimal contacts in individual patients. The model matched the clinician's choice in determining the optimal stimulation level in 71% of cases. By testing only the predicted level rather than all possible options, programming time could be substantially reduced. For conventional stimulation with 4 contacts per electrode, this corresponds to a 75% reduction in the number of tests (4–1/4). For directional stimulation with 8 segmented contacts, testing only the predicted segment reduces the number of tests from 8 to 3 on average, corresponding to 62.5% reduction (8–3/8). These estimates are in line with previous reports showing that algorithmic or image‐guided DBS programming approaches can shorten programming time by 25% to 75%.^32^, ^33^, ^34^

This is particularly remarkable for segmented electrode designs, where contact distances are comparable in size to the imaging resolution, even when models were originally trained on heterogeneous group‐level data. In fact, this heterogeneity in the training data might be considered a strength rather than a limitation, potentially contributing to the model's success in suggesting optimal contacts in our prospectively acquired patients. Evidence from other fields suggests that identifying brain stimulation targets based on heterogeneous data from different modalities will prevent overfitting and lead to robust models that will more likely generalize to novel data.^35^ This matches our results, where circular and CV correlations were similar, indicating a low risk of overfitting, and the similar amount of variance explained in the “noisy” training and test datasets. Moreover, the successful identification of the optimal stimulation contact suggests that these models trained to explain variance between‐subjects in fact explain more meaningful variance within‐subjects. Instead, when using the same models to predict clinical outcomes following DBS in “noisy” cohorts, electrode placement remains key but other factors seem at least equally important.

Whereas the combined ridge regression model improved predictive performance compared to any univariate model, the absolute explained variance in the validation cohort remained modest. This aligns with the expected limits of predictability in heterogeneous multicenter datasets, in which electrode placement represents only one of many factors determining treatment success. Importantly, ridge regression coefficients represent conditional rather than standalone importance for each predictor. For example, although DBS Fiber Filtering received the highest weight within the joint model, it showed poor performance when tested in isolation. This discrepancy likely reflects the colinear nature of imaging‐derived predictors and the fact that each of these predictors contribute complementary information.

We note that the LMEs analysis revealed a slope <1, indicating underestimation of absolute improvement magnitudes. Although this slope provides a useful summary of the correspondence between predicted and empirical improvements, the primary source of this compression arises from the regularized ridge regression used to generate the predictions. Ridge regression inherently shrinks coefficients toward zero, compressing predicted values toward the mean and reducing the predicted dynamic range. This shrinkage improves robustness and prevents overfitting, particularly in heterogeneous datasets with multiple sources of variability. Crucially, the model still preserves the relative ranking of contacts within patients, which is the clinically relevant information for guiding DBS programming, so this underestimation does not reduce its practical utility.

Our results confirm optimal target sites for STN‐DBS on the coordinate, sweet spot, tract, and network levels and are directly relevant for clinical practice. Both the optimal coordinate and sweet spot of our model pointed to the Bejjani line,^36^ widely used for surgical targeting in clinical practice. Moreover, the optimal coordinates and sweet spots precisely matched multiple published reports.^37^, ^38^ At the tract and network modalities, our identified connections are in agreement with most historical and modern previous reports.^39^, ^40^ Whereas confirmatory to published reports, given the large cohort of patients, our result may represent a “mature” model of optimal stimulation targets on 5 modalities of analysis whose performance has now been properly quantified and used to suggest stimulation contacts with a high success rate in unseen data. These results lay the groundwork for applying our model in prospective clinical trials that could ultimately save time for both clinicians and patients, making DBS programming more efficient, accurate, and deliberate.

We selected ridge regression as the primary technique for combining the 5 modality‐specific predictors because it directly addresses the multicollinearity inherent in DBS imaging models, which all rely on shared electrode localization and therefore have strongly correlated surrogate variables. Ordinary multiple linear regression is unstable under such conditions, whereas ridge penalization shrinks correlated coefficients toward one another and yields more robust parameter estimates without performing variable selection. We deliberately avoided least absolute shrinkage and selection operator (LASSO) or elastic‐net penalization, as the goal of the present study was not feature selection and eliminating predictors would not be desirable. In our case, each modality represents a theoretically motivated and clinically interpretable dimension of DBS effects. We also did not apply more complex machine‐learning approaches, such as support vector regression, random forests, and neural networks. These methods generally require larger training datasets to avoid overfitting, and their nonlinear models are substantially less interpretable than linear penalized regression. Dimensionality‐reduction approaches, such as principal component regression, were also deemed less suitable because they reduce interpretability by transforming predictors into components that no longer correspond to the specific modeling approaches that we aimed to compare. Given our emphasis on interpretability, stability, and generalizability to unseen datasets, including a prospective cohort, ridge regression offered the best balance of performance and transparency.

Our study is subject to multiple limitations. First, the model was created based on retrospective heterogeneous data from multiple institutions. These cohorts differed in terms of post‐surgical data collection times, clinical centers, electrode models, neurosurgeons, neurologists, and countries. However, as argued above, this choice was deliberate to maximize robustness and prevent overfitting to a single surgeon or center.^35^ Second, none of the datasets analyzed contained information about side effects. This information could certainly increase the clinical utility of our model, because for now, it is making decisions only based on motor effects. Third, for this line of work, nonlinear registrations between native patient and template space are required, which, despite decade‐long improvements of the underlying methodology, will always introduce a certain amount of bias. To minimize this bias, we used a state‐of‐the‐art DBS imaging pipeline designed and optimized for DBS imaging analyses.^1^ Fourth, biophysical modeling and sweet spot statistics may be carried out in various ways, and whereas our study tested several methodological concepts, it would not be possible to compare every single method that has been proposed over the years.^41^ Last, whereas it is standard practice to mirror stimulation volumes across hemispheres in DBS mapping,^9^, ^10^, ^11^, ^42^, ^43^ this approach cannot fully resolve the inherent asymmetry between bilateral stimulation sites and a single global outcome measure, and residual dependency between hemispheres may influence voxel‐wise estimates. These limitations reflect a broader methodological constraint of DBS outcome mapping and underscore the need for future work developing truly lateralized clinical metrics or unilateral stimulation paradigms to more directly disentangle hemisphere‐specific effects.

In conclusion, we introduce a new multimodal DBS image guidance model which explained a significant amount of variance in 2 validation datasets and suggested clinically sensible electrode contact choices. By computationally estimating the clinical impact of each contact within an individual patient, it may potentially guide clinicians toward the most optimal contacts for initial DBS programming, reducing both the duration and burden of the process and improving clinical practice.

## Author Contributions

P.Z., N.R., and A.H. contributed to the conception and design of the study; P.Z., N.R., K.B., C.V.L., T.B., J.N.P.‐S., T.A.D., M.T.B., V.V.‐V., M.R., J.V., V.J.J.O., R.M.A.B., X.X., Z.L., C.Y., A.A.K., N.L., G.M.M., and A.R.P. contributed to the acquisition and analysis of data; P.Z., N.R., A.H., M.D.F., G.M.M., K.B., I.A.S., B.H.H., L.L.G., S.R.S., K.R., and B.H. contributed to drafting and significant portion of the manuscript or figures.

## Potential Conflicts of Interest

The authors do not report conflicts of interest related to this work.

## Supporting information


**Supplementary Data S1:** Supplementary Information.

## Data Availability

All code used to analyze the dataset is openly available within Lead‐DBS (including sweet spot mapping, fiber filtering, and network mapping software: https://github.com/leaddbs/leaddbs). Cohort‐wise demographic and clinical outcomes from the training and the test cohorts are available in Supplementary Tables S2 and S3. We cannot openly share patient imaging data due to data sharing and privacy regulations, but they can be made available upon request to the corresponding primary investigator. The corresponding author and the principal investigator (P.Z. and A.H.) commit to returning data requests within a time frame of 30 days.

## References

[1] Neudorfer C , Butenko K , Oxenford S , et al. Lead‐DBS v3.0: mapping deep brain stimulation effects to local anatomy and global networks. Neuroimage 2023;268:119862. 10.1016/j.neuroimage.2023.119862. 36610682 PMC10144063

[2] Lozano AM , Lipsman N , Bergman H , et al. Deep brain stimulation: current challenges and future directions. Nat Rev Neurol 2019;15:148–160. 10.1038/s41582-018-0128-2. 30683913 PMC6397644

[3] Behnke JK , Peach RL , Habets JGV , et al. Long‐term stability of spatial distribution and peak dynamics of subthalamic Beta power in Parkinson's disease patients. Mov Disord 2025;40:1070–1084. 10.1002/mds.30169. 40099366 PMC12160969

[4] Busch JL , Kaplan J , Bahners BH , et al. Local field potentials predict motor performance in deep brain stimulation for Parkinson's disease. Mov Disord 2023;38:2185–2196. 10.1002/mds.29626. 37823518

[5] Roediger J , Dembek TA , Wenzel G , et al. StimFit—A data‐driven algorithm for automated deep brain stimulation programming. Mov Disord 2022;37:574–584. 10.1002/mds.28878. 34837245

[6] Roediger J , Dembek TA , Achtzehn J , et al. Automated deep brain stimulation programming based on electrode location: a randomised, crossover trial using a data‐driven algorithm. The Lancet Digital Health 2023;5:e59–e70. 10.1016/S2589-7500(22)00214-X. 36528541

[7] Miocinovic S , Noecker AM , Maks CB , et al. Cicerone: stereotactic neurophysiological recording and deep brain stimulation electrode placement software system. Acta Neurochir Suppl 2007;97:561–567. 17691348 10.1007/978-3-211-33081-4_65

[8] Maks CB , Butson CR , Walter BL , et al. Deep brain stimulation activation volumes and their association with neurophysiological mapping and therapeutic outcomes. J Neurol Neurosurg Psychiatry 2009;80:659–666. 10.1136/jnnp.2007.126219. 18403440 PMC2859444

[9] Rajamani N , Friedrich H , Butenko K , et al. Deep brain stimulation of symptom‐specific networks in Parkinson's disease. Nat Commun 2024;15:4662. 10.1038/s41467-024-48731-1. 38821913 PMC11143329

[10] Hollunder B , Ostrem JL , Sahin IA , et al. Mapping dysfunctional circuits in the frontal cortex using deep brain stimulation. Nat Neurosci 2024;27:573–586. 10.1038/s41593-024-01570-1. 38388734 PMC10917675

[11] Horn A , Reich M , Vorwerk J , et al. Connectivity predicts deep brain stimulation outcome in Parkinson disease. Ann Neurol 2017;82:67–78. 10.1002/ana.24974. 28586141 PMC5880678

[12] Li N , Baldermann JC , Kibleur A , et al. A unified connectomic target for deep brain stimulation in obsessive‐compulsive disorder. Nat Commun 2020;11:3364. 10.1038/s41467-020-16734-3. 32620886 PMC7335093

[13] Ríos AS , Oxenford S , Neudorfer C , et al. Optimal deep brain stimulation sites and networks for stimulation of the fornix in Alzheimer's disease. Nat Commun 2022;13:7707. 10.1038/s41467-022-34510-3. 36517479 PMC9751139

[14] Reich MM , Horn A , Lange F , et al. Probabilistic mapping of antidystonic effect of pallidal neurostimulation: multicentre imaging study. Brain 2019;142:1386–1398. 10.1093/brain/awz046. 30851091

[15] Meyer GM , Hollunder B , Li N , et al. Deep brain stimulation for obsessive‐compulsive disorder: optimal stimulation sites. Biol Psychiatry 2023;96:101–113. 10.1016/j.biopsych.2023.12.010. 38141909 PMC11190041

[16] Alonso F , Zsigmond P , Wårdell K . Influence of Virchow‐Robin spaces on the electric field distribution in subthalamic nucleus deep brain stimulation. Clin Neurol Neurosurg 2021;204:106596. 10.1016/j.clineuro.2021.106596. 33813373

[17] Avants BB , Tustison NJ , Song G , et al. A reproducible evaluation of ANTs similarity metric performance in brain image registration. Neuroimage 2011;54:2033–2044. 10.1016/j.neuroimage.2010.09.025. 20851191 PMC3065962

[18] Fonov V , Evans AC , Botteron K , et al. Unbiased average age‐appropriate atlases for pediatric studies. Neuroimage 2011;54:313–327. 10.1016/j.neuroimage.2010.07.033. 20656036 PMC2962759

[19] Oxenford S , Ríos AS , Hollunder B , et al. WarpDrive: improving spatial normalization using manual refinements. Med Image Anal 2024;91:103041. 10.1016/j.media.2023.103041. 38007978 PMC10842752

[20] Horn A , Li N , Dembek TA , et al. Lead‐DBS v2: towards a comprehensive pipeline for deep brain stimulation imaging. Neuroimage 2019;184:293–316. 10.1016/j.neuroimage.2018.08.068. 30179717 PMC6286150

[21] Husch A , V. Petersen M , Gemmar P , et al. PaCER ‐ a fully automated method for electrode trajectory and contact reconstruction in deep brain stimulation. Neuroimage Clin 2017;17:80–89. 10.1016/j.nicl.2017.10.004. 29062684 PMC5645007

[22] Horn A , Kühn AA . Lead‐DBS: a toolbox for deep brain stimulation electrode localizations and visualizations. Neuroimage 2015;107:127–135. 10.1016/j.neuroimage.2014.12.002. 25498389

[23] Dembek TA , Hoevels M , Hellerbach A , et al. Directional DBS leads show large deviations from their intended implantation orientation. Parkinsonism Relat Disord 2019;67:117–121. 10.1016/j.parkreldis.2019.08.017. 31495733

[24] Butenko K , Bahls C , Schröder M , et al. OSS‐DBS: open‐source simulation platform for deep brain stimulation with a comprehensive automated modeling. PLoS Comput Biol 2020;16:e1008023. 10.1371/journal.pcbi.1008023. 32628719 PMC7384674

[25] Baldermann JC , Melzer C , Zapf A , et al. Connectivity profile predictive of effective deep brain stimulation in obsessive‐compulsive disorder. Biol Psychiatry 2019;85:735–743. 10.1016/j.biopsych.2018.12.019. 30777287

[26] Poldrack RA , Huckins G , Varoquaux G . Establishment of best practices for evidence for prediction: a review. JAMA Psychiatry 2020;77:534–540. 10.1001/jamapsychiatry.2019.3671. 31774490 PMC7250718

[27] Sobesky L , Goede L , Odekerken VJJ , et al. Subthalamic and pallidal deep brain stimulation: are we modulating the same network? Brain 2022;145:251–262. 10.1093/brain/awab258. 34453827

[28] van der Linden C , Berger T , Brandt GA , et al. Accelerometric classification of resting and postural tremor amplitude. Sensors (Basel) 2023;23:8621. 10.3390/s23208621. 37896714 PMC10611060

[29] van der Linden C , Berger T , Dembek TA , et al. Tractography‐guided versus clinical contact selection for deep brain stimulation in tremor ‐ a prospective clinical trial. Brain Stimul 2026;19:103061. 10.1016/j.brs.2026.103061. 41740836

[30] Horn A . The impact of modern‐day neuroimaging on the field of deep brain stimulation. Curr Opin Neurol 2019;32:511–520. 10.1097/WCO.0000000000000679. 30844863

[31] Hines K , Noecker AM , Frankemolle‐Gilbert AM , et al. Prospective Connectomic‐based deep brain stimulation programming for Parkinson's disease. Mov Disord 2024;39:2249–2258. 10.1002/mds.30026. 39431498 PMC11659031

[32] Pavese N , Tai YF , Yousif N , et al. Traditional trial and error versus neuroanatomic 3‐dimensional image software‐assisted deep brain stimulation programming in patients with Parkinson disease. World Neurosurg 2020;134:e98–e102. 10.1016/j.wneu.2019.09.106. 31568905

[33] Pourfar MH , Mogilner AY , Farris S , et al. Model‐based deep brain stimulation programming for Parkinson's disease: the GUIDE pilot study. Stereotact Funct Neurosurg 2015;93:231–239. 10.1159/000375172. 25998447

[34] Lange F , Steigerwald F , Malzacher T , et al. Reduced programming time and strong symptom control even in chronic course through imaging‐based DBS programming. Front Neurol 2021;12:12. 10.3389/fneur.2021.785529. PMC8606823 34819915

[35] Siddiqi SH , Kording KP , Parvizi J , Fox MD . Causal mapping of human brain function. Nat Rev Neurosci 2022;23:361–375. 10.1038/s41583-022-00583-8. 35444305 PMC9387758

[36] Bejjani BP , Dormont D , Pidoux B , et al. Bilateral subthalamic stimulation for Parkinson's disease by using three‐dimensional stereotactic magnetic resonance imaging and electrophysiological guidance. J Neurosurg 2000;92:615–625. 10.3171/jns.2000.92.4.0615. 10761650

[37] Akram H , Sotiropoulos SN , Jbabdi S , et al. Subthalamic deep brain stimulation sweet spots and hyperdirect cortical connectivity in Parkinson's disease. Neuroimage 2017;158:332–345. 10.1016/j.neuroimage.2017.07.012. 28711737 PMC6581538

[38] Bot M , Schuurman PR , Odekerken VJJ , et al. Deep brain stimulation for Parkinson's disease: defining the optimal location within the subthalamic nucleus. J Neurol Neurosurg Psychiatr 2018;89:493–498. 10.1136/jnnp-2017-316907. 29353236

[39] Treu S , Strange B , Oxenford S , et al. Deep brain stimulation: imaging on a group level. Neuroimage 2020;219:117018. 10.1016/j.neuroimage.2020.117018. 32505698

[40] Hassler R , Riechert T , Mundinger F , et al. Physiological observations in stereotaxic operations in extrapyramidal motor disturbances. Brain 1960;83:337–350. 10.1093/brain/83.2.337. 13852002

[41] Dembek TA , Baldermann JC , Petry‐Schmelzer JN , et al. Sweetspot mapping in deep brain stimulation: strengths and limitations of current approaches. Neuromodulation 2022;25:877–887. 10.1111/ner.13356. 33476474

[42] Butenko K , Neudorfer C , Dembek TA , et al. Engaging dystonia networks with subthalamic stimulation. Proc Natl Acad Sci 2025;122:e2417617122. 10.1073/pnas.2417617122. 39773021 PMC11745339

[43] Dembek TA , Roediger J , Horn A , et al. Probabilistic sweet spots predict motor outcome for deep brain stimulation in Parkinson disease. Ann Neurol 2019;86:527–538. 10.1002/ana.25567. 31376171

