# Supplementary material for: Multimodal Image Guidance in Subthalamic Deep Brain Stimulation for Parkinson's Disease
Source: Ann Neurol. 2026 Apr 17;100(1):22–35. doi: 10.1002/ana.78206 (PMC13327559; doi:10.1002/ana.78206)
Supplement: Supplementary file 1 — Supplementary Data S1: Supplementary Information. [file ANA-100-22-s001.docx]

**Supplementary Material for: Multimodal Image guidance in Subthalamic Deep Brain Stimulation for Parkinson’s Disease**

Patricia Zvarova^1,2,3,4^, Christina van der Linden^5^, Ningfei Li^1^, Konstantin Butenko^1^, Thea Berger^5^, Garance M. Meyer^3^, Ilkem Aysu Sahin^1,2,4^, Lukas L. Goede^1,3^, Bahne H. Bahners^3,6,7^, Barbara Hollunder^1,2,8^, Till A. Dembek^5^, Andrew R. Pines^3,9^, Martin Reich^10^, Jens Volkmann^10^, Vincent J.J. Odekerken^11^, Rob M. A. de Bie^11^, Xin Xu^12^, Zhipei Ling^13^, Chen Yao^14^, Andrea A. Kühn^1,2,8,15,16^, Surjo R. Soekadar^2,17^, Kerstin Ritter^2,18,19^, Michael T. Barbe^5^, Veerle Visser-Vandewalle^20^, Michael D. Fox^3^, Jan Niklas Petry-Schmelzer^5^, Nanditha Rajamani^1,3†^, Andreas Horn^3, 4, 21†^

^†^These authors contributed equally to this work.

Table of Contents

[Supplementary Text S1 3](#_Toc224583967)

[Supplementary Table S1 7](#_Toc224583968)

[Supplementary Figure S1 8](#_Toc224583969)

[Supplementary Methods S1 9](#_Toc224583970)

[Supplementary Methods S2 12](#_Toc224583971)

[Supplementary Methods S3 14](#_Toc224583972)

[Supplementary Figure S2 16](#_Toc224583973)

[Supplementary Table S2 17](#_Toc224583974)

[Supplementary Figure S3 17](#_Toc224583975)

[Supplementary Figure S4 18](#_Toc224583976)

[Supplementary Table S3 19](#_Toc224583977)

[Supplementary Table S4 20](#_Toc224583978)

[Supplementary Figure S5 21](#_Toc224583979)

[Supplementary Results S1 22](#_Toc224583980)

[Supplementary Figure S6 23](#_Toc224583981)

[Supplementary Table S5 25](#_Toc224583982)

[Supplementary Figure S7 26](#_Toc224583983)

[Supplementary Figure S8 26](#_Toc224583984)

[Supplementary Results S2 28](#_Toc224583985)

[Supplementary Figure S9 29](#_Toc224583986)

[Supplementary Table S6 31](#_Toc224583987)

[Supplementary Figure S10 32](#_Toc224583988)

[Supplementary References 33](#_Toc224583989)

# Supplementary Text S1

**Factors contributing to clinical improvements in STN-DBS for Parkinson’s disease**

Before developing models aimed at (1) explaining clinical variance in group level data and (2) suggesting optimal contacts in individual patients, we began with theoretical considerations and literature review to inform expectations for both tasks.

To inform the estimates shown in Figure 1, we conducted a focused, narrative literature search aimed at identifying factors reported to influence clinical motor outcomes after STN-DBS in Parkinson’s disease. Because only a minority of studies explicitly quantify variance explained by individual predictors, and no standardized taxonomy exists for reporting them, we used a pragmatic, expert-informed approach. First, PubMed and Google Scholar were searched using combinations of terms (“Parkinson’s disease”, “deep brain stimulation”, “predictors”, “outcome”, “STN”, “variance explained”, “clinical improvement”). Second, senior coauthors with longstanding expertise in DBS clinical research (MR, JV, VJJO, RMAdB, AAK, MTB, VVV, MDF, AH) were asked to identify additional studies they considered informative but that might not have surfaced through keyword search alone. Studies were included if they reported any empirical association between patient-, disease-, anatomical-, imaging-, or stimulation-related factors and DBS motor outcomes, even if the reported effect sizes were indirect (e.g., standardized coefficients, between-group contrasts, or qualitative associations). When exact variance estimates were unavailable (as was the case for most studies) we derived conservative, lower-bound approximations based on the reported statistics. Because heterogeneous methods, outcome definitions, and covariate structures precluded a formal meta-analysis, the resulting contributions in Figure 1 should be interpreted as literature-informed *estimates* rather than quantitative effect sizes. This process reflects the current state of the field, in which systematic and directly comparable predictor analyses are still lacking.

One of the most comprehensive studies was conducted by Cavallieri et al.^1^, who analyzed predictors of long-term outcomes in patients with PD who underwent DBS in a long-term cohort of 138 patients and short-term cohort of 357 patients. The study assessed the contribution of ten different predictors: Age at the surgery, onset age, young onset Parkinson’s disease (YOPD), presence of white matter hyperintensities on brain MRI, Mattis Dementia Rating Scale (MDRS), Frontal Score, part III of the Movement Disorders Society Unified Parkinson’s Disease Rating Scale (MDS-UPDRS) off-medication before the surgery, Hoehn & Yahr stage off-medication, levodopa responsiveness and tremor dominant phenotype (TDP). Apart from TDP, all factors contributed significantly to the short-term cohort. In the long-term cohort, significant contributors were frontal lobe dysfunction, disease severity, and the presence of vascular changes. Since the variance explained by these predictors (and their covariance structure) was not reported, we estimated the contributions based on the reported standardized ß-coefficients (Reference 5 in Figure 1A and the Supplementary Table 1). Other studies that we reported in a category termed “disease state” focused on the contribution of anxiety and depression (Reference 11 in Figure 1A and Supplementary Table 1).^2^ This study reported the variance explained in DBS-related improvement, which we rounded down to avoid inflated effects. The idea that emotional well-being affects DBS outcomes is not new, but most evidence remains anecdotal.^3^

Beyond disease state, anatomical abnormalities, especially near the target nucleus, can influence clinical response to DBS. For example, microstructural changes such as the presence of Virchow-Robin spaces near the electrode influence the spread of electric field beyond the target nucleus (Reference 9 in Figure 1A and Supplementary Table 1).^4^ Neither image guidance models nor most biophysical modeling approaches account for these structural abnormalities in individual patients. As a result, predicting clinical outcomes for individual contacts in such patients may yield imprecise results. Besides microstructural abnormalities, cortical atrophy present in paracentral areas and superior frontal cortex may lead to decreased responsiveness of DBS (Reference 10 Figure 1A and Supplementary Table 1).^5^ If we enter two patients with an ideal (and equal) electrode placement within the STN into our model, the model would predict the same optimal improvement for both. However, if one of these patients had substantial atrophy in frontal areas, clinical effects would likely be less optimal in this patient compared to the other one.

Although the UPDRS-III is well established and healthcare providers are well trained to assess the motor symptoms of PD, there is always an expected error between different clinical assessments (be it the intra- or the inter-rater error). Studies that looked into this issue^6–9^ reported excellent reliability based on non-significant statistical group differences. However, small margins of error were observed. For these reasons, we did not exclude the small variance explained by these two factors, but we decided to keep it rather low at 2% for both intra- and inter-rater reliability, respectively. It is important to note that our clinical outcomes were assessed across various DBS centers, countries and continents (References 1-4 in Figure 1A and Supplementary Table 1).

Since our analysis focused on image guidance, model performance depends on how imaging data was collected, processed and analyzed. Estimating the variance explained by our selection of neuroimaging methods is not straightforward, since we use a combination of different methods that have not been compared head-to-head. Further, various methods have been proposed to approximate stimulation effects^10,11^ (references 5 and 6 in Figure 1A and the Supplementary Table 1). In our methods, we make assumptions about the physical properties of the electric field imposed by the electrode, the conductivity of axons, as well as the intactness of the brain. Moreover, inaccuracies in electrode reconstruction in both the patient’s native space and standard stereotactic space may increase modeling uncertainty (reference 7 in Figure 1A and the Supplementary Table 1). Rajamani et al.^12^ examined how adding uncertainty (jitter) to their electrode location affected model performance based on optimal stimulation tracts. Although the exact amount of variance introduced was not reported, the average spatial correlation between their baseline model and 1,000 jittered models was 0.80. Based on these assumptions, we estimated approximately 10% of variance explained to be attributed to the neuroimaging factor. We visualize all aforementioned factors in Figure 1A.

While the earlier considerations addressed explaining variance in clinical improvement using group-level data, the nuisance variables differ substantially when the goal is to suggest optimal contacts for individual patients. The reason is that here, most of the abovementioned variables would be fixed, i.e. would not contribute noise within the individual patient. For instance, the patient will have the same age, PD subtype, comorbidities, etc. when our question is which of their electrode contacts would perform the best. We still identified three categories that would represent nuisance variables in individual patients, which are inaccuracy introduced by imaging, microstructural anatomical changes (which may have differential effects on individual electrode contacts) and a high covariance structure in clinical effects across neighboring contacts.

Imaging (References 6-8 in Figure 1B and Supplementary Table 1)^10–12^ and anatomical factors (Reference 9 in Figure 1B and Supplementary Table 1)^4^ were already discussed previously (Figure 1A). As we are still analyzing neuroimaging data, the processing pipeline may introduce uncertainty, and anatomical deviations could further affect the model’s accuracy. Microstructural anatomical changes within the target region such as structural lesions or Virchow-Robin spaces could contribute to unaccounted noise since they may differentially impact the clinical response of certain electrode contacts, but not others. The third factor, clinical measures, addresses that stimulating adjacent electrode contacts will often lead to similar effects, and it may not always be straightforward to identify a single contact that is clearly the best, especially when dealing with segmented electrode designs. Factors such as testing sequence, patient fatigue, and intra-rater variability can make the identification of the “optimal contact” somewhat imprecise and subjective, even in meticulously and prospectively acquired data, as in the present study. In our models, which are based on anatomical positions, no two contacts will ever be assigned identical values. This is because each contact is always located in a unique anatomical position, so the model will assign (slightly) distinct values for each contact within a given patient. With these considerations, we estimate that, while the electrode contact choice could theoretically account for all the variance, image-guided models should optimally only be able to account for around 70% of the variance when suggesting optimal contacts for individual patients. We visualize all these factors in Figure 1B. It is also important to note, that imaging resolution is relatively low and capturing within-contact differences at this level of precision is still quite astonishing (Figure 1C).

Complete list of references from Figure 1: [1-4]^6–9^, [5]^1^, [6-8]^10–12^, [9]^4^, [10]^5^, [11]^2^

Supplementary Table S1. Factors contributing to clinical improvement after deep brain stimulation (reference table).

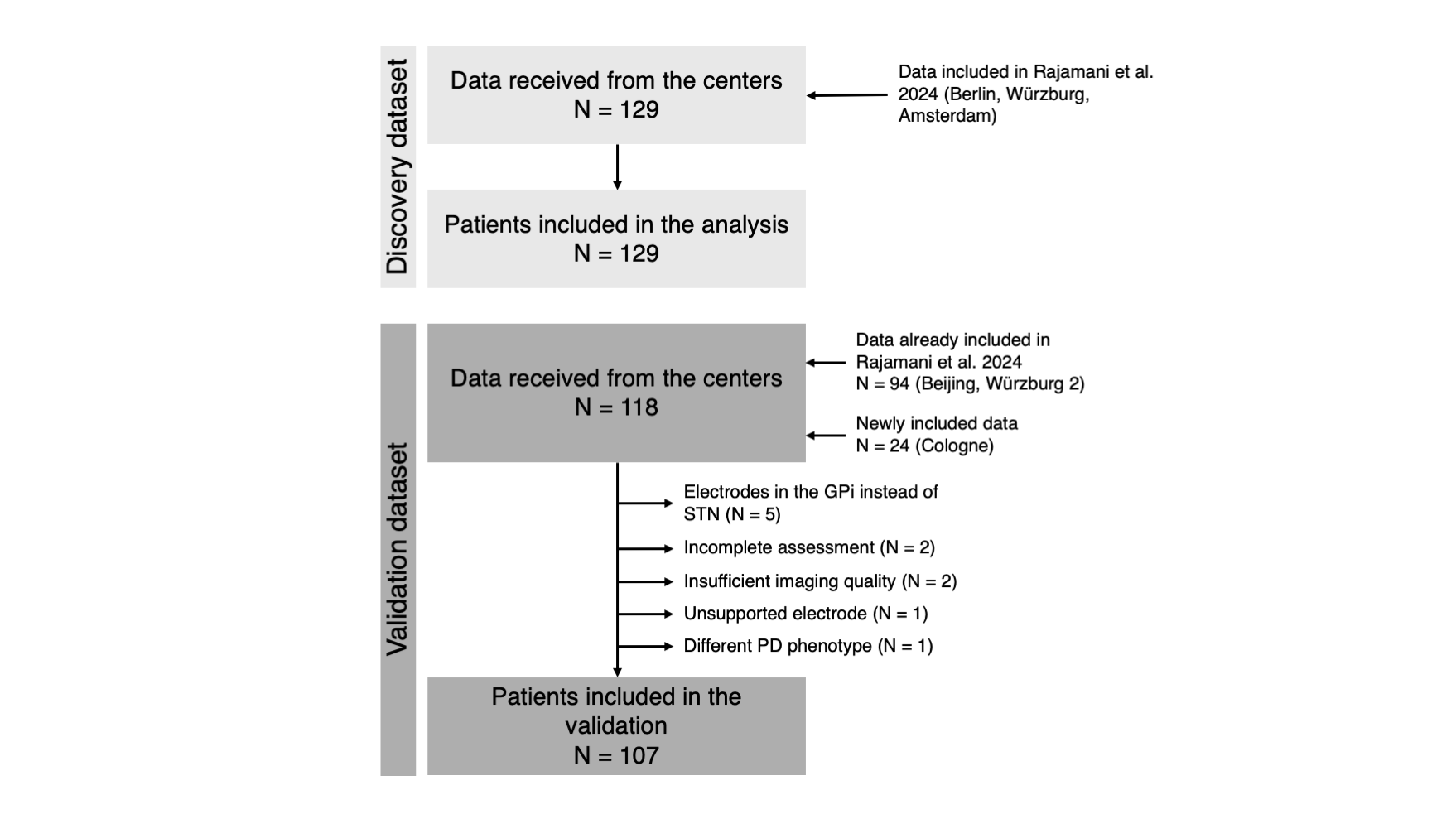


Supplementary Figure S1: Flowchart of inclusion and exclusion criteria.

# Supplementary Methods S1

**Extended DBS modeling methods**

In this study, we conceptualized the five modeling approaches as distinct modalities of analysis, each representing an increasing spatial and conceptual scale. The analyses progress from local, site-specific models within the STN (proximity and sweet spot mapping) to fiber-modality and whole-brain network models (fiber filtering, structural, and functional connectivity). The first modality included the optimal active contact target, expressed as x-, y- and z-coordinates in MNI (MNI152NLin2009bAsym template) space (millimeters). After electrode localizations were transformed into a normative space, we extracted the 3-dimensional coordinates of patient-specific contacts selected for chronic stimulation. If multiple contacts per patient were stimulated, the coordinates of these contacts were averaged to obtain a single location per patient. To determine the stimulation coordinates associated with optimal clinical improvements, coordinates were weighted by the 10^th^ power of normalized patient-specific relative UPDRS-III improvements. The 10^th^ power was chosen to maximize differences between high and low responders, giving greater weight to patients with higher clinical improvement. The resulting coordinates were averaged to yield a single point in 3D space.

For the second modality, we explored the stimulation profile associated with the optimal clinical improvements in a DBS Sweet Spot analysis.^13,14^ E-fields estimated for each patient based on their stimulation parameters were thresholded at 100 V/m. Only voxels included in the stimulation volumes of at least 10% of patients were retained. Global UPDRS-III improvement scores were assigned to each hemispheric stimulation volume, reflecting the fact that clinical motor outcomes were assessed under bilateral stimulation. Since no asymmetries of DBS effects on motor symptoms are to be expected^3,13,15,16^, and as done in prior DBS mapping studies^3,12,17–20^ stimulation fields were mirrored to the contralateral side to increase spatial sampling and to align all stimulation sites within a common anatomical reference frame. This mirroring step also accounts for ipsilateral stimulation effects, which can contribute to clinical improvement but are not captured when analyses are restricted to the original unilateral volume. Thus, each voxel contained 258 values associated with the E-field magnitudes from the discovery dataset. These values were correlated with clinical improvements, resulting in a profile of voxels associated with optimal (sweet spot) or suboptimal (sour spot) clinical improvement. While sweet spot voxels were defined by voxels that positively correlated with the clinical improvement, sour-spot voxels were simply defined by voxels negatively correlated with the clinical improvement. Only significant voxels (*p* < 0.05) were considered for the model.

For the third modality, we explored the association between streamlines and clinical improvements using the DBS Fiber Filtering method.^12,13^ The Netstim Atlas V2.2^12^ was used to represent streamline candidates in the STN region. Like the sweet spot modality, we did not expect lateralization of DBS motor effects based on global UPDRS-III scores, therefore streamlines were mirrored across hemispheres to maximize statistical power of the model. E-field values associated with tract “activations” are typically considered to be in the range of above ~200 V/m.^21^ To unify the thresholding strategies between DBS Sweet Spot mapping and DBS Fiber Filtering, while still avoiding spurious correlations, we applied a less conservative threshold and discarded streamlines that were not covered by E-field magnitudes of at least 100 V/m in any patient. For similar reasons, we also discarded streamlines that traversed the periphery of electric fields in all patients, i.e., were not clearly modulated by at least a few electrodes. Of the remaining streamlines, only those covered by an E-field magnitude of at least 500 V/m in more than 3% of all stimulation fields were retained for the final model. For the main analysis, thresholds for DBS Sweet Spot and DBS Fiber Filtering analysis were unified; however, additional thresholds at the level of 50 V/m, 150 V/m, 200 V/m were also tested (see Supplementary Figures 7 and 8).

To determine the optimal streamline profile associated with the maximum DBS related clinical benefit, for each streamline we correlated the patient-specific E-field magnitude posed on the streamline with the patient-specific clinical outcomes. Positive correlations indicated streamlines associated with greater clinical improvement, while negative correlations indicated streamlines associated with less optimal outcomes.

For the fourth and fifth modalities, we applied the DBS Network Mapping approach using both structural and functional connectomes^3^ to estimate cortico-subcortical connectivity, based on voxel-wise correlations. Structural networks were defined using the same atlas as in the fiber-filtering step.^12^ Functional networks were defined using resting-state functional MRI (rs-fMRI) from 1,087 scans in the Human Connectome Project^22^ stored in a matrix format. To estimate the structural DBS connectivity profile seeding from each patient’s stimulation volumes, we calculated the overlap between voxels with the E-fields information and the voxels with the streamlines of the normative structural connectome. To estimate the functional connectivity, we first binarized the E-fields at a typical threshold value of 200 V/m.^21^ Functional connectivity profiles for each patient were then calculated as described in Goede et al.^23^ In short, we used a precalculated whole brain voxel-wise connectome based on 1,000 resting-state fMRI scans (four runs each) acquired within the Human Connectome Project^22^. This average voxel-wise connectome is stored as a large adjacency matrix in 2 mm isotropic resolution (i.e. storing the connectivity of each voxel in MNI space). To obtain functional connectivity profiles seeding from each patient’s stimulation volumes, the rows of the connectome corresponding were averaged to obtain a whole-brain profile. This procedure was repeated for each patient to acquire their unique functional connectivity fingerprint.

Optimal network profiles for both structural and functional data were calculated using the combined map (C-map) approach, which has shown robust performance in previous work.^3^ These maps represent the connectivity profile of stimulation volumes, that are positively associated with clinical outcomes.

Namely, first, fingerprint maps were correlated with clinical improvement using Pearson’s correlations, resulting in a correlation map (R-map). Next, weighted average maps were calculated by averaging all fingerprints weighted by their respective clinical improvements (A-map). Finally, the R-maps and the A-maps were combined by retaining the A-map voxels with the same sign as the corresponding R-map, resulting in the final C-map.^3^

# Supplementary Methods S2

**Surrogate variable calculation**

For each patient, we generated surrogate estimates of clinical improvement using multiple neuroimaging-based models. At the coordinate modality, the surrogate estimates were calculated as the Euclidean distance from the optimal stimulation site.

$d=\sqrt{\left( x_{1}-x_{2} \right)^{2}+\left( y_{1}-y_{2} \right)^{2}+\left( z_{1}-z_{2} \right)^{2}}$ (Eq. 1)

Where *x_1_, y_1_,* and *z_1_* represent coordinates of the optimal stimulation site, and *x_2_, y_2_,* and *z_2_* represent the coordinates of the active electrode contact, calculated as the contact centroid.

For the remaining modalities (E-field, tract, structural network, and functional network) these surrogate estimates were computed as spatial similarity measures (Pearson correlation coefficients) between the patient-specific data, and the corresponding “optimal” model derived from the discovery dataset. The Pearson correlation was calculated as:

$r=\frac{\text{Cov}\left( X,Y \right)}{\sigma_{X}\sigma_{Y}}$ (Eq. 2)

Where *X* represents patient-specific data vector and *Y* represents the model-derived “optimal” vector. Thus, *Y* is fixed, but *X* varies between patients. Each correlation value represents how closely a patient’s stimulation pattern matches the model-defined optimal pattern, serving as a quantitative surrogate for the expected clinical response (and hence represents our estimates). These five surrogate values were then used as predictors in a ridge regression model to integrate information across modalities.

At the E-field modality, spatial similarity between each E-field and the sweet spot model was calculated using Pearson’s correlation as done in previous studies.^13,24,25^ Here, the surrogate variable for the clinical improvement was expressed as the spatial correlation coefficient between sweet spots and electric fields. The intuitive interpretation of this is as follows: if a patient’s E-field profile spatially resembles the landscape defined as “optimal” by the sweet spot, the model would expect high clinical improvement. Ten-fold cross-validation was carried out analogously for the coordinate, E-field, tract, and network modalities.

At the tract modality, the surrogate was expressed similarly to the sweet spot model, i.e., by measuring the overlap between E-field magnitudes of a given patient with the optimal streamline model. The same interpretation applies: if the shape of an electric field precisely matched the one defined by the optimal tract landscape, the model would estimate higher DBS response. After performing both in-sample evaluation and ten-fold cross-validation, we correlated the surrogate with the empirical clinical improvement, expecting consistently positive correlation results.

Finally, on a network modality, the surrogate was defined using the same metric: each patient’s network profile (connectivity fingerprint) was spatially correlated with the optimal stimulation network profile. If the spatial definition of the network profile seeding from the patient’s E-fields matched the optimal DBS network profile, the model would estimate optimal DBS response, resulting in higher surrogate values. Again, we applied both in-sample evaluation and ten-fold cross-validations.

Across these models (coordinates, E-fields, tracts, structural and functional networks), each patient of the discovery dataset was assigned five surrogate values (essentially representing similarities between each patient and the model) determined from the in-sample design. These values were later used to fit a ridge regression model, which provided beta estimates for the out-of-sample validation in the hold-out cohort.

To quantify how well each method predicted the contact-wise clinical responses, we fitted a separate general linear model (GLM) for each method, using the contact-level surrogate value as the predictor and the observed clinical improvement as the dependent variable. These GLMs were estimated independently for each electrode, ensuring that comparisons reflected within-electrode ranking performance rather than between-patient variability. For electrode *j* and contact *i*, the model can be written as:

$\text{Empirical }\text{Improvement}_{ij}=\beta_{0}^{\left( j \right)}+\beta_{1}^{\left( j \right)}\cdot\text{Predicted}_{ij}+\varepsilon_{ij}$ (Eq. 3)

Where *ß_0_* is the intercept for electrode *j*, *ß_1_* is the slope, and ε is the residual error for contact *i* of electrode *j*. Model fit was summarized using the Pearson correlation coefficient between predicted and observed contact-wise improvements, and these correlations were aggregated across electrodes to quantify average performance per method.

# Supplementary Methods S3

**Ridge regression regularization parameter calculation**

To determine a suitable ridge regression regularization parameter (*λ* factor), we followed guidelines provided by MathWorks (<https://www.mathworks.com/help/stats/ridge.html>), where the *λ* factor is referred to as the variable *k*. Critically, this process was carried out exclusively using the discovery cohort, before any attempts were made to explain variance in the hold-out test dataset. Ridge regression minimizes the following objective function:

$\hat{\beta^{ridge}}=\arg\min_{\beta} \left\{ \sum_{i=1}^{n} \left( y_{i}-X_{i}\beta\right)^{2}+\lambda\sum_{j=1}^{p} \beta_{j}^{2} \right\}$ (Eq. 4)

Where $y_{i}$ represents the empirical outcome, $X_{i}$ represents the predictors (surrogate values), $ß$ represents the regression coefficient and $\lambda$(or *k* in MATLAB) is the regularization parameter that shrinks coefficients toward zero to stabilize estimates. In MATLAB, the ridge function can be fitted as *B = ridge (y, X, k, 0)*, where *y* is the clinical improvement as an outcome variable, *X* is the matrix containing our surrogate values, k corresponds to the ridge regularization parameter and the *0* indicates that the intercept is not automatically removed from the regression.

Following the recommended guidelines, we first tested how different *k* values affected coefficient estimates. To do so, we created a matrix of predictors, including the interaction terms. The interaction terms were only included in the ridge trace to visualize the stability of coefficient estimates as a function of *λ* values. By nature, interaction terms contain more noise and therefore serve as a reliable sensitive test of regression stability, showing when even the noisiest regressors stabilize. We then plotted the standardized coefficients obtained during in-sample cross-validation against the *k* parameter. The goal was for the model to stabilize, i.e., for the standardized coefficients to converge and for the plotted lines to flatten. We tested three different *k* coefficients (*k* = 10, 50, and 90) on our model from the discovery dataset to assess changes in the correlation between estimated and empirical clinical improvement, as well as changes in beta estimates (Supplementary Figure 2A). The resulting plots suggested that the overall model predictions did not substantially change based on the selected ridge parameters (Supplementary Figure 2B).

Preliminary exploration of the ridge regression model suggests that standardized coefficients changed only minimally when *λ* was varied in increments of 10 across the range of 1 – 100. Based on this observation, we focused subsequent nested cross-validation analysis on *λ* values of 10 to 100, selecting the optimal *λ* in a quantitative fashion. The dataset was divided into five outer folds (5-fold CV) and three inner folds (3-fold CV). In each outer iteration, one-fold was held out as the test set and the remaining four folds were used for training. Within each outer training set, we performed three-fold cross-validation to select optimal *λ* value from the list of those 10 values. For each *λ* candidate, a ridge regression model was trained on two folds of the inner training set and evaluated on the left-out fold. The mean squared error (MSE) was calculated and averaged across inner folds, and the *λ* value that yielded the lowest MSE was selected as the optimal for the outer fold. This *λ* value was then applied to the entire training set of the outer fold and then tested on the held-out outer fold. Test set performance was calculated using MSE and *R^2^*. Across outer folds, the *λ* values ranged from 30 to 50 (Supplementary table 2), with the median of 50. The mean inner fold *R^2^* as a function of *λ* (Supplementary Figure 3) showed a plateau, with performance peaking at *λ* = 50. Given the stable coefficients across this range, *λ* = 50 was chosen for the final model.

The robustness of ß estimates of the final model was tested using ten-fold cross-validation (Supplementary Figure 4A) and bootstrapping (Supplementary Figure 4B). In the ten-fold analysis, we split the training dataset into ten folds and iteratively estimated ß using nine folds for training and one for validation. This yielded 10 different ß estimates per method. For bootstrapping, we resampled the training dataset with replacement over 1000 iterations, calculating ß estimates in each run.

The in-sample scores were used to build a model to maximize coefficient stability, but the model was also tested via ten-fold cross-validation and bootstrapping. All modeling considerations and model training were based exclusively on discovery cohort. Only the final models for each modality, as well as the combined model were tested on the hold-out test datasets (N = 89 and N = 21).


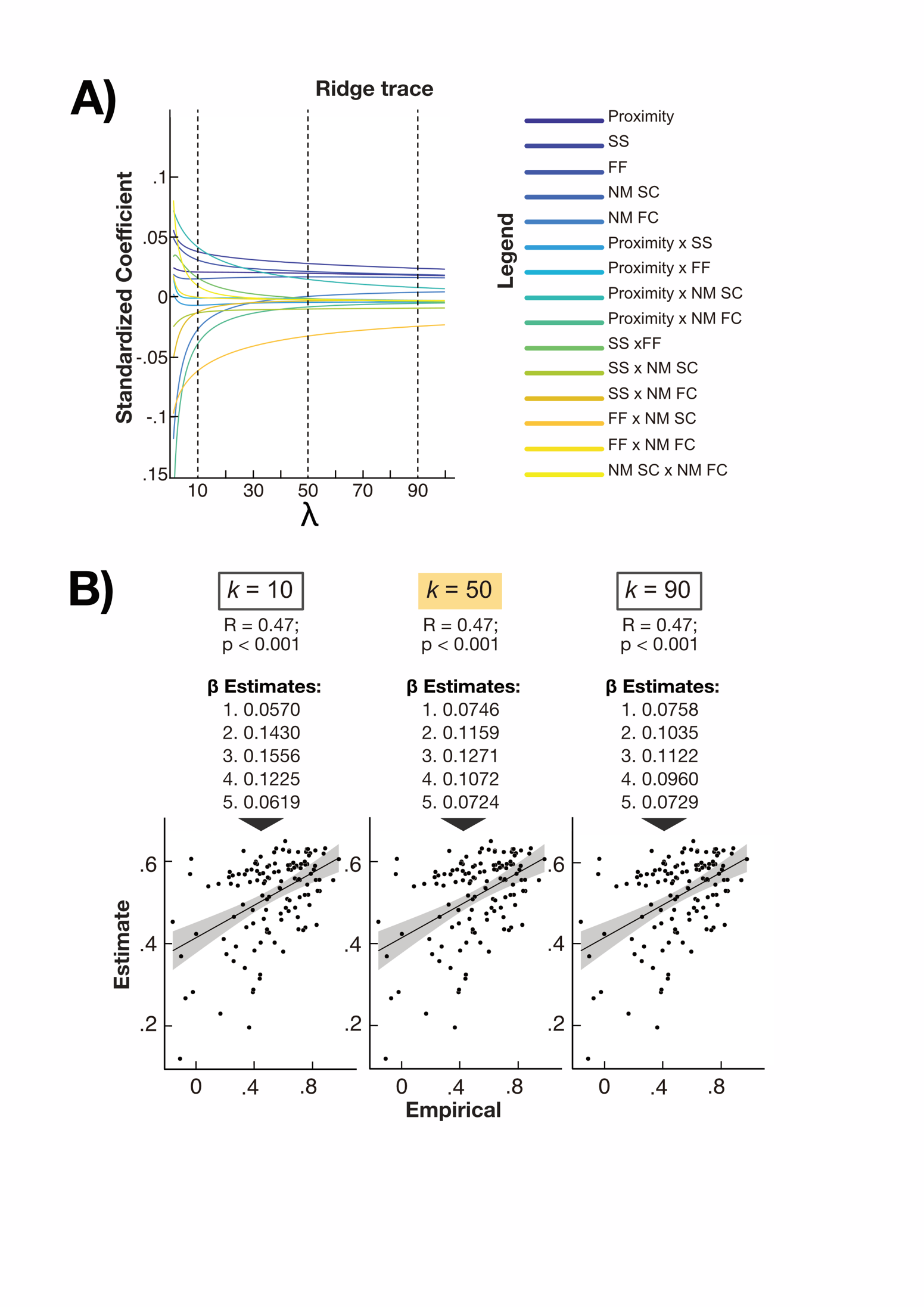


**Supplementary Figure S2. Ridge parameter (k) determination.** Five different predictors and their interaction terms were used to fit the ridge regression. We tested 100 different values to determine the ridge parameter. A) A plot of estimates for predictors and interaction terms using 100 different ridge parameters (*k*). Three parameters were tested to plot the ridge regression (*k* = 10, *k* = 50, *k* =90). The correlation of the predicted values and beta estimates for each of the ridge parameter tested is reported. The ridge parameter 50 (highlighted in yellow) was selected for the final model. B) Correlation plots between estimated values and the empirical values based on the ridge regression model with three different *k* parameters (*k* = 10, *k* = 50, *k* =90). SS – DBS Sweet Spot Mapping surrogate estimate, FF – DBS Fiber Filtering surrogate estimate, NM SC – DBS Network Mapping surrogate estimate, NM FC – DBS Network Mapping surrogate estimate

**Supplementary Table S2.** Results of the outer loop of nested CV

| Iteration | MSE | R2 | Chosen *λ* |
| --- | --- | --- | --- |
| 1 | 0.02 | -0.05 | 50 |
| 2 | 0.05 | 0.13 | 30 |
| 3 | 0.06 | 0.23 | 50 |
| 4 | 0.05 | 0.17 | 40 |
| 5 | 0.06 | 0.17 | 50 |


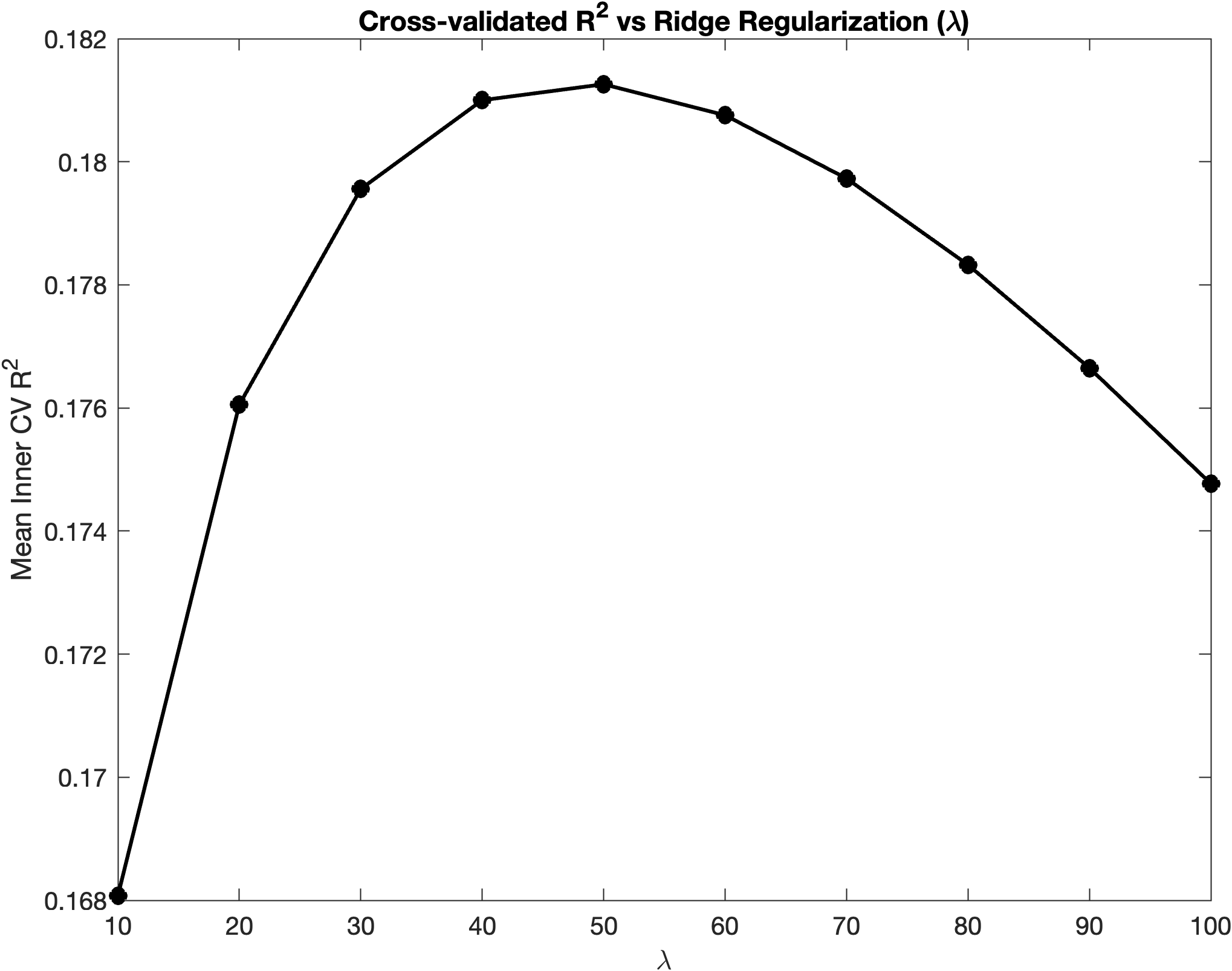


**Supplementary Figure S3. Average prediction performance of the inner loop of nested cross-validation for each tested regularization parameter (*λ*).** The ridge regression regularization parameter was selected based on model performance in nested cross-validation. The average variance explained (R^2^) across inner folds is plotted as a function of λ. Model performance peaked at λ = 50 (R^2^ = 0.18) and λ = 50 was retained as the regularization parameter for the final model.

**
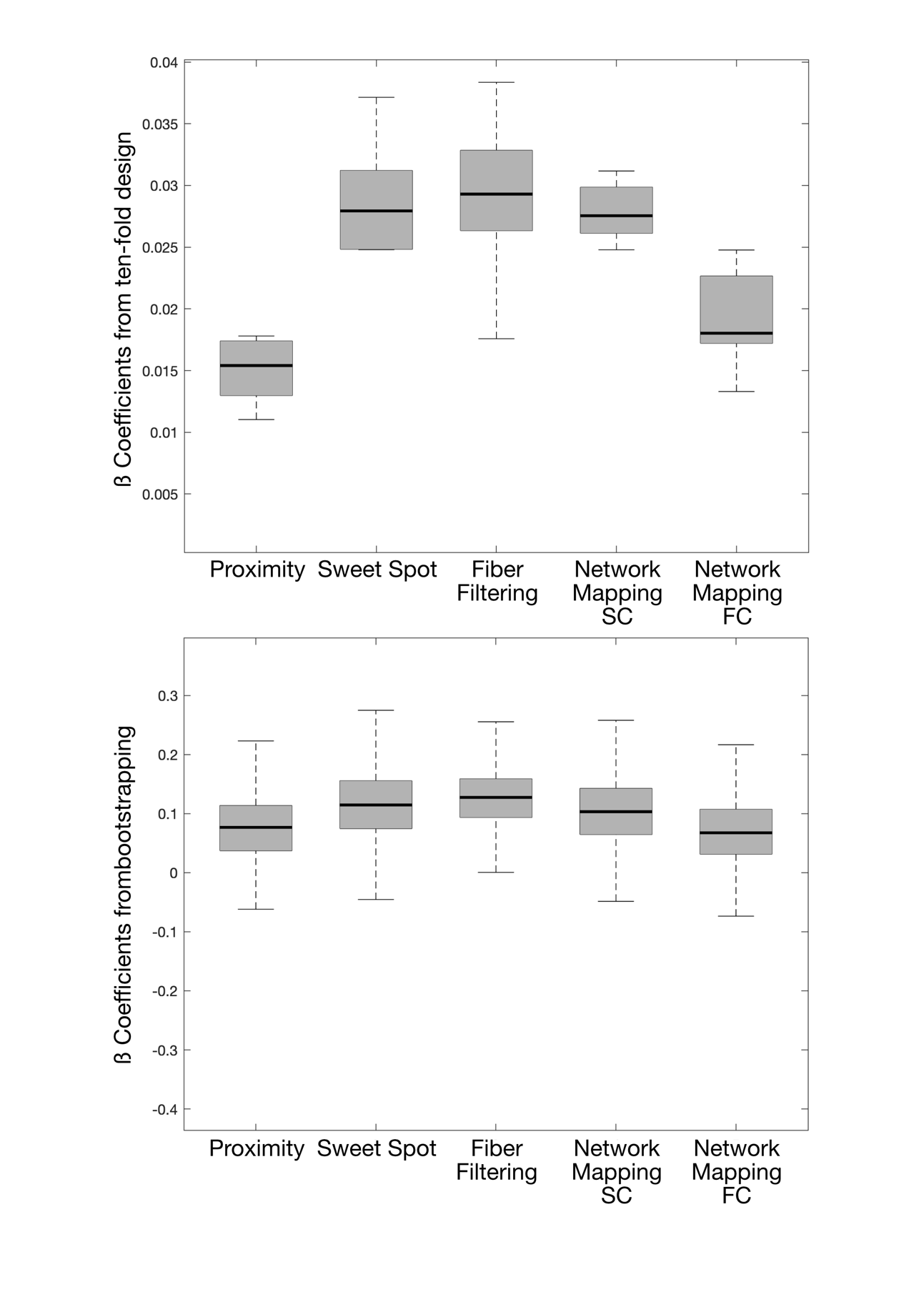
**

**Supplementary Figure S4. Distribution of ß estimates calculated on the training dataset using ten-fold validation and bootstrapping.** Two techniques were used to test the stability of ß estimates of our model. Black lines represent the mean, boxes represent interquartile range, whiskers represent the most extreme data points, and + sign represents the outliers. A) Our dataset was split into ten-folds and ß estimates were calculated by fitting ridge regression always on nine out of ten folds. Ten different ß estimates were calculated for each method. B) Bootstrapping with resampling method was used to calculate ß estimates in 1000 iterations. SC – structural connectivity, FC – functional connectivity.

**Supplementary Table S3**: Demographics of the discovery and test dataset

| **Discovery** **Cohort** | | | |
| --- | --- | --- | --- |
| **Center** | Berlin | Würzburg | Amsterdam |
| **Demographic information** | | | |
| **Surgical DBS Center** | Charité - Universitäts-medizin Berlin | University Hospital Würzburg | Department of Neurology, Amsterdam University Medical Center, Amsterdam, Netherlands |
| **N (female)** | 51 (17) | 44 (12) | 34 (7) |
| **Age at time of surgery (mean ± SD; range; in years)** | 60 ± 8 | 60 ± 8 | 50 ± 10 |
| **Clinical Details** | | | |
| **Average time of follow-up (in months)** | 40 | N/A | N/A |
| **UPDRS-III scores at baseline (STIM-OFF/MED-OFF) (mean ± SD)** | 38.56 ± 12.92 | 49.41 ± 12.54 | 47.03 ± 15.53 |
| **UPDRS-III scores at time of follow-up under stimulation ON condition (STIM-ON/MED-OFF) (mean ± SD)** | 20.12 ± 8.82 | 24.47 ± 10.62 | 15.07 ± 10.43 |
| **Rel. improvement (mean ± SD; in %)** | 45.34 ± 23.03 | 49.46 ± 23.97 | 64.13 ± 22.40 |
| **Abs. improvement (mean ± SD)** | 18.44 ± 11.31 | 25.37 ± 13.30 | 31.06 ± 14.97 |
| **Levodopa Response (mean % ± SD)** | 53 ± 42 | 61 ± 25 | 45 ± 34 |
| **Imaging and DBS Specification** | | | |
| **Imaging modality (post-operatively)** | MRI/CT | CT | CT |
| **Electrode models** | Medtronic 3389 | Medtronic 3389 | Medtronic 3389 |
| **Related citation** | ^3,12^ | ^3,12^ | ^12^ |

**Supplementary Table S4**: Information about the retrospective (Beijing & Würzburg) and prospective (Cologne) test datasets.

| **Validation (Hold-Out Test) Datasets** | | | |
| --- | --- | --- | --- |
| **Center** | Beijing | Würzburg | Cologne |
| **Demographic information** | | | |
| **Surgical DBS Center** | Chinese PLA General Hospital | University Hospital Würzburg | Department of Neurology, University of Cologne |
| **N (female)** | 41 (21) | 53 (19) | 24 |
| **Age at time of surgery (mean ± SD; in years)** | 61 ± 10 | 60 ± 8 | 58 ± 8 |
| **Clinical Details** | | | |
| **Time of follow-up (in months)** | N/A | 11 | N/A |
| **UPDRS-III scores at baseline (STIM-OFF/MED-OFF) (mean ± SD)** | 45.73 ± 13.12 | 42.53 ± 10.78 | 10.26 ± 3.45^a^ |
| **UPDRS-III scores at time of follow-up under stimulation ON condition (STIM-ON/MED-OFF) (mean ± SD)** | 19.17 ± 10.76 | 22.45 ± 10.04 | 5 ± 3 |
| **Rel. improvement (mean ± SD; in %)** | 58.01 ± 21.29 | 46.38 ± 10.04 | 79.27 ± 14.96^b^ |
| **Abs. improvement (mean ± SD)** | 26.56 ± 12.86 | 20.07 ± 10.91 | 8.07 ± 2.76^b^ |
| **Levodopa Response (mean % ± SD)** | N/A | N/A | N/A |
| **Imaging and DBS Specification** | | | |
| **Imaging modality (post-operatively)** | CT | CT | CT |
| **Electrode models** | Medtronic 3389 | Boston Scientific Vercise Directed/Boston Scientific Vercise | Medtronic B3305/Boston Scientific Vercise Directed |
| **Related citation** | ^12^ | ^12^ | ^26^ |

**^a^** Only a subset of UPDRS-III items per one hemisphere was assessed, including: Upper Extremity Rigidity, Finger Tapping, Resting Tremor, Postural Tremor, and Kinetic Tremor.

**^b^** Absolute and relative (%) improvements in this case were calculated based on the contact with the highest clinical response.


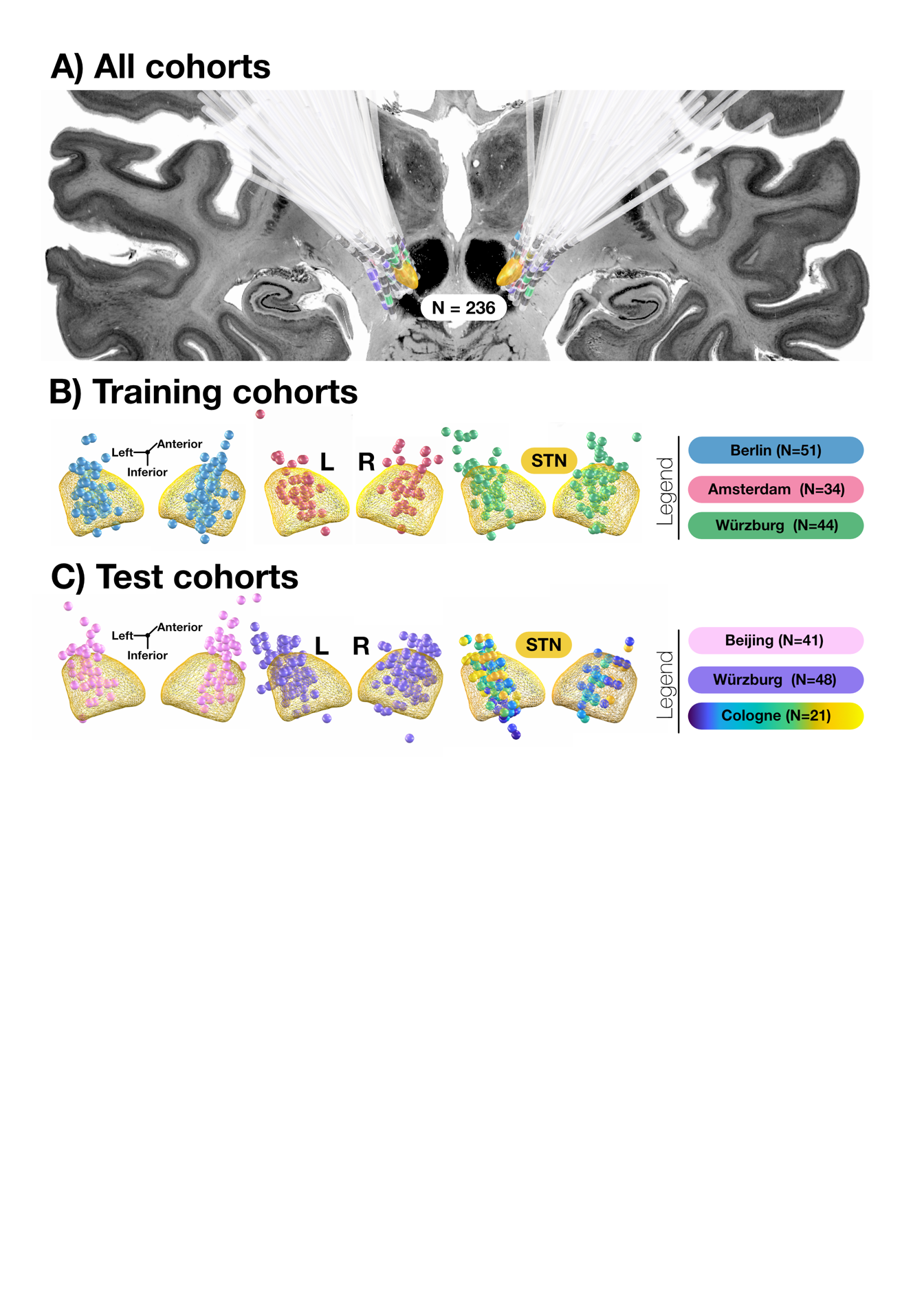


**Supplementary Figure S5. Distribution of electrode placements relative to the subthalamic nucleus (STN) across cohorts.** Electrode reconstructions across all cohorts relative to the STN (as defined by the DISTAL atlas^27^) are shown. DBS electrode placements were reconstructed using Lead-DBS software. The training cohort consisted of 129 patients who underwent bilateral DBS using omnidirectional electrodes with four stimulating contacts (Medtronic 3389), targeting the STN. The retrospective validation dataset consisted of 89 patients from two PD cohorts, implanted with three types of electrodes: Medtronic 3389 and Boston Scientific Vercise Directed. The prospectively acquired validation cohort consisted of 18 patients (21 electrodes) implanted with Boston Scientific Vercise Directed electrodes and one patient with Medtronic B3305 electrode. A) Electrodes (N = 457) from all patients included in the study are shown. A coronal slice at -20.0 mm of the BigBrain template,^28^ in 100-µm resolution is visualized in the background for anatomical reference. Active contacts from patients in the training (B) and test (C) datasets are visualized as spheres relative to the STN. Since data from 8 contacts were collected for each patient in the prospectively acquired validation dataset, each patient was assigned a unique color, and the eight spheres representing their active contacts were color-coded accordingly.

# Supplementary Results S1:

**Data-driven imaging models of clinical improvement**

Each of our models was compared to previously published results in the literature. At the coordinate modality, our optimal target coordinate was in very close proximity to, and slightly anterior to, the one published by Caire et al., 2013 (1.37 mm distance; Supplementary Figure 6A).^29,30^ At the E-field modality, our sweet spot model peaked at a similar location with the peak aligning with both the Bejjani line^31^ often used for surgical targeting in clinical practice, and previously published reports (Supplementary Figure 6B).^15,32–34^ At the tract modality, projections from Brodmann areas (BA) 4, 6, 8 to the STN, as well as sensorimotor connections between the STN and the pallidum, and connections between the STN and the pedunculopontine nucleus (PPN), were associated with optimal clinical improvement (Supplementary Figure 6C). A table listing these connections is provided in Supplementary Table 4, where the results generally match prior reports.^12,32^ Applying different DBS Sweet Spot and DBS Fiber Filtering thresholds for E-field inclusion led to comparable results (Supplementary Figure 7 and Supplementary Figure 8). At the whole-brain network modality, both structural and functional DBS network mapping analyses were carried out following previously reported approaches.^3^ For structural connectivity, optimal clinical improvements were associated with connections between the STN and both the SMA and pre-SMA (Supplementary Figure 6D), consistent with previously published findings.^3,15^ For functional connectivity, positive connectivity to frontal and inferior parietal regions, as well as negative connectivity with the primary motor and visual cortices, were associated with optimal response (Supplementary Figure 6E), again consistent with previously published findings.^3,15^


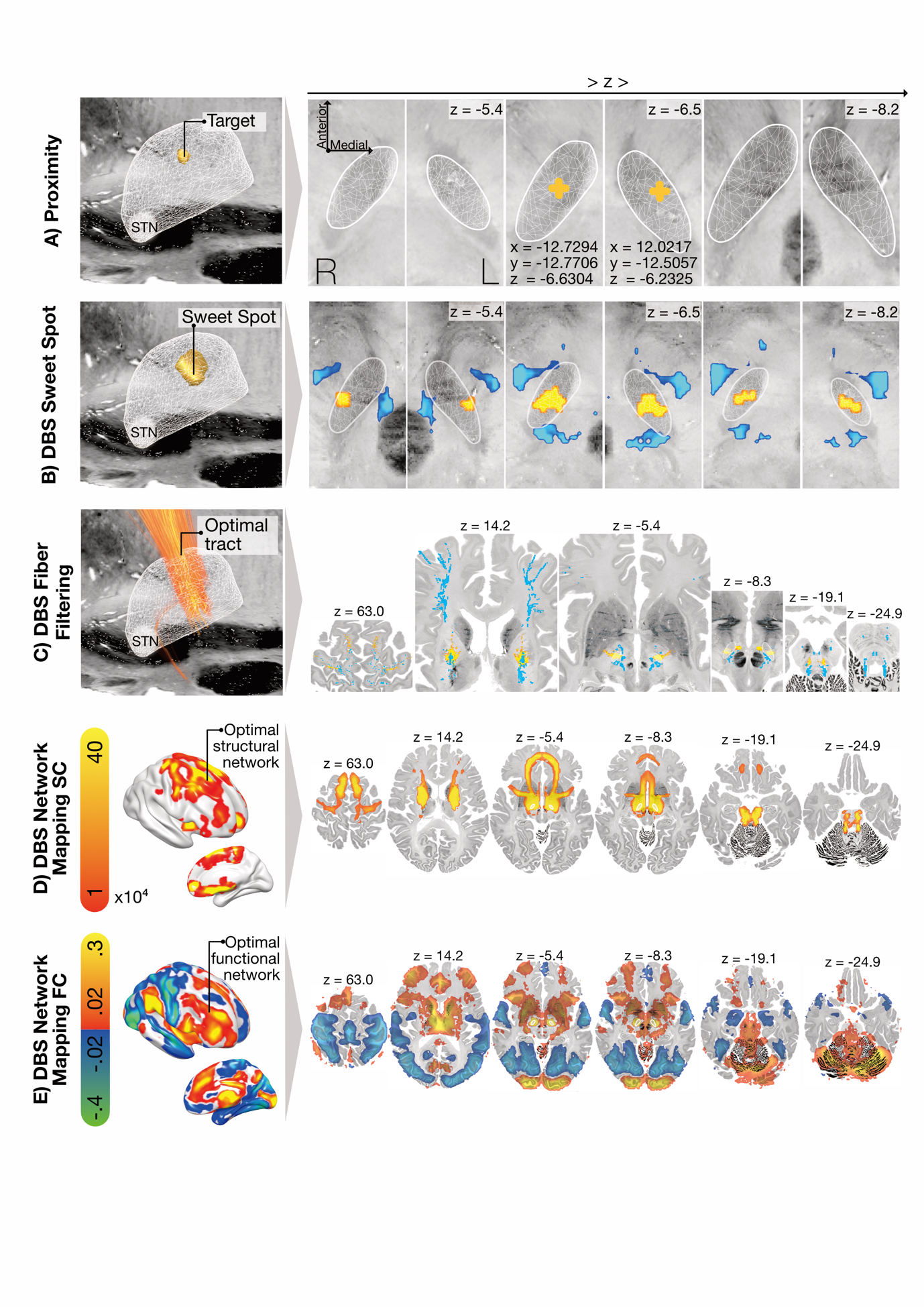


**Supplementary Figure S6. Anatomical representation of data-driven neuroimaging models of clinical improvement in Parkinson’s Disease (PD) following deep brain stimulation (DBS).** Five distinct methods were used to associate clinical improvement with electrode position at different modalities. For each model, the electrode position was obtained using Lead-DBS software. The BigBrain template^28^ in 100-µm resolution is visualized in the background for anatomical reference. Gold indicates regions associated with clinical improvement. Blue indicates regions associated with less optimal outcomes. White mesh structures represent the subthalamic nucleus (STN). A) The optimal stimulation coordinate was defined as the weighted average of active contact locations, with weights based on clinical improvements (golden cross). Exact x, y, z coordinates are reported for the MNI152 NLin2009bAsym template space, which is the default space of Lead-DBS. B) The sweet spot was calculated using voxel-wise correlations between clinical improvements and electric field magnitudes. The resulting maps were thresholded at the uncorrected significance level (α = 0.05). C) Optimal tract connections were calculated using a manually curated, population-based pathway atlas (Netstim Atlas V2.2) via DBS fiber filtering.^12^ The optimal streamline profile was modeled by correlating peak electric field magnitudes along each streamline with clinical improvements. D) The same pathway atlas was used to calculate an optimal structural connectivity profile using a voxel-wise fashion (DBS network mapping). Voxels were assigned values based on the stimulation volumes overlapping the streamlines. The combined map approach was used to determine the optimal connectivity profile, as proposed in ^3^. E) The optimal functional connectivity profile was calculated from resting-state functional MRI (rs-fMRI) data from 1,087 scans in the Human Connectome Project. Seeding from the stimulation volumes, network connectivity maps were computed and correlated with clinical improvement using the same combined map approach.

**Supplementary Table S5**. DBS Fiber tracts from the Netstim Atlas V2.2 that correlated (positively and negatively) with UPDRS-III clinical improvement. The anatomical names of tracts are listed in the descending order based on their number of streamlines associated with the model.

| **No.** | **Positive streamlines** | **Negative streamlines** |
| --- | --- | --- |
| **1.** | STN to Brodman area 6 connections | Dentatorubrothalamic tract (drtt-SMA) |
| **2.** | STN to Brodman area 4 connections | STN to Brodman area 1, 2, 3 connections |
| **3.** | STN to Brodman area 8 connections | STN to Brodman area 4 connection |
| **4.** | STN to Pallidum connection | STN to Brodman area 10 connections |
| **5.** | STN to pedunculopontine nucleus connections | STN to Brodman area 45 and 47 connections |
| **6.** | STN to Brodman area 10 connections | Pedunculopontine nucleus to SMA connections |
| **7.** | STN to Brodman area 1, 2, 3 connections | Rubrocortical tract  Corticospinal tract |
| **8.** | Corticospinal tract | Rubrocortical tract |
| **9.** |  | \| Dentatorubrothalamic tract (drtt-M1) \| \| --- \| |
| **10.** |  | \| STN to Brodman area 6 connections \| \| --- \| |
| **11.** |  | STN - Pallidum |
| **12.** |  | \| STN to Brodman area 8 connections \| \| --- \| |


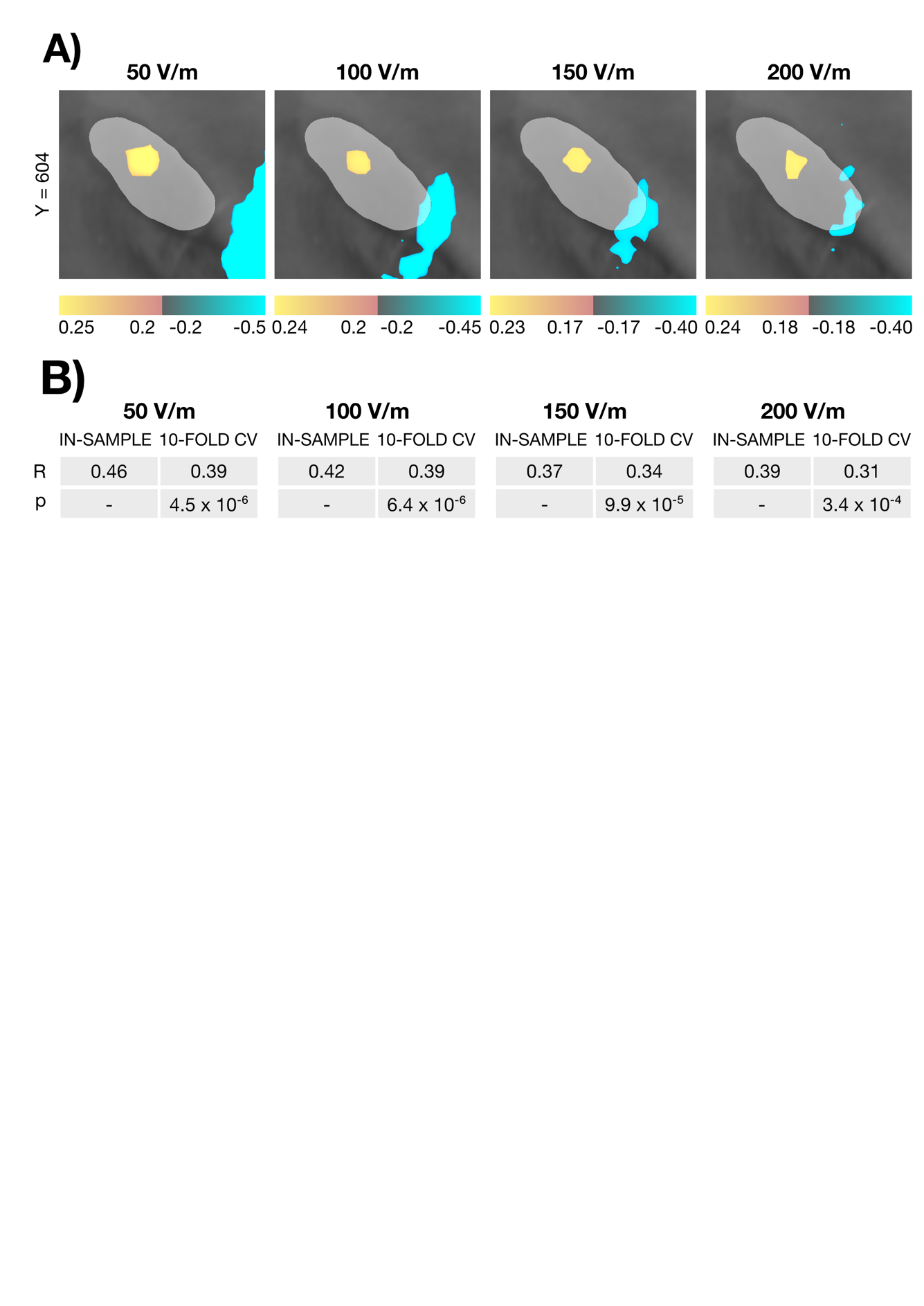


**Supplementary Figure S7. DBS Sweet Spot analysis results using different electric field (E-field) thresholds.** Electrodes were reconstructed using Lead-DBS and electric fields were calculated using OSS-DBS v2. E-fields were then thresholded using four different thresholds: 50 V/m, 100 V/m, 150 V/m, 200 V/m. In each case, E-field values below the set threshold were not included in the analysis. The remaining E-field values were used to calculate the sweet spot using voxel-wise correlations between their magnitudes and clinical improvements. The resulting maps were thresholded at the uncorrected significance level (α = 0.05). A) Visualization of resulting sweet- and sour-spot values in an MNI152 NLin2009bAsym template space using 7T image of an ex-vivo human brain as a background ^35^. B) Validation of DBS Sweet Spot results for different electric field thresholding values using circular (in-sample) and ten-fold analysis.


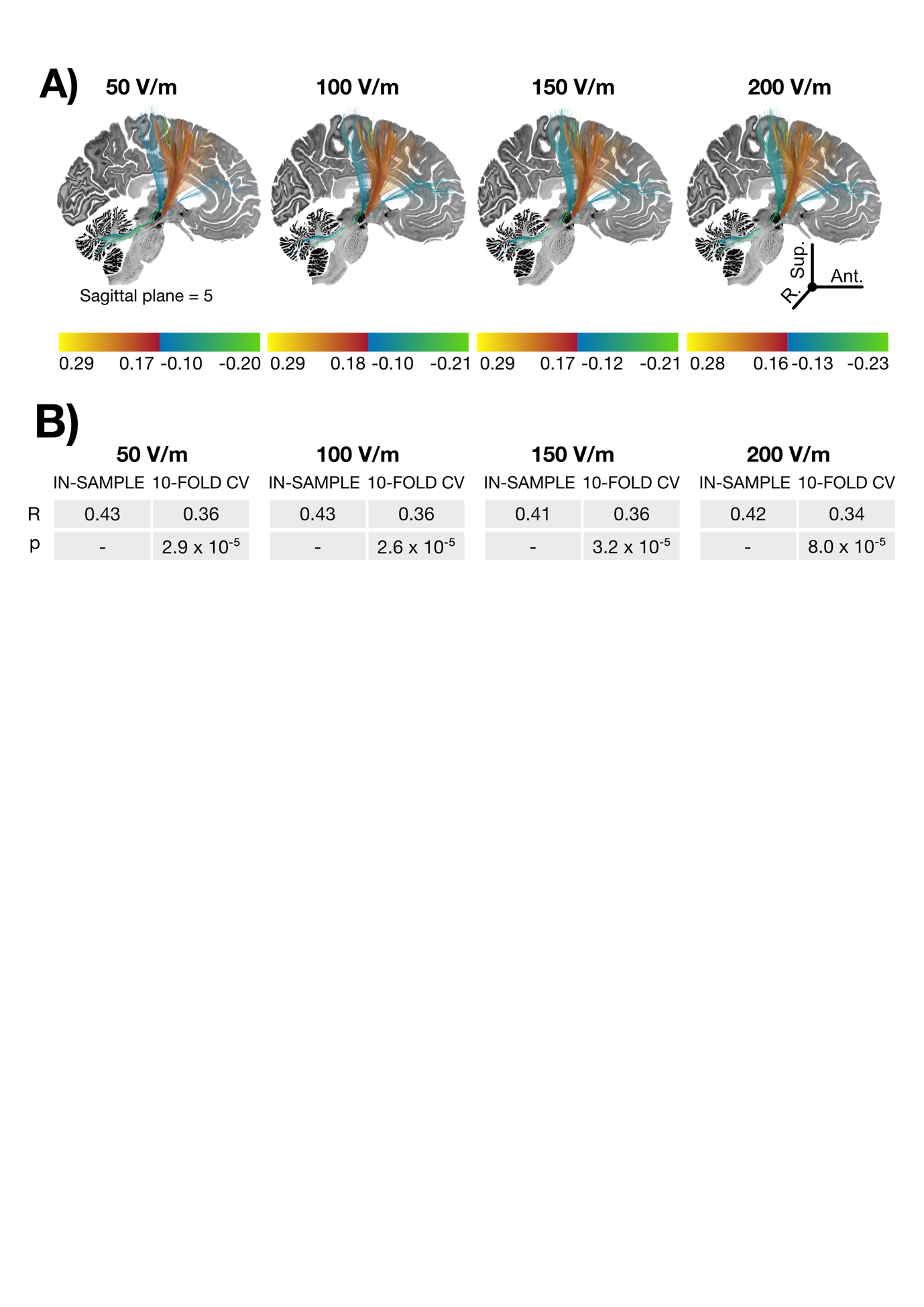


**Supplementary Figure S8. DBS Fiber Filtering analysis results using electric field (E-field)-based thresholding of streamline inclusion.** Electrodes were reconstructed using Lead-DBS and electric fields were calculated using OSS-DBS v2. E-fields were then thresholded using four different thresholds: 50 V/m, 100 V/m, 150 V/m, 200 V/m. In each case, streamlines passing electric fields below the magnitude of the set threshold were not included in the analysis. Resulting streamlines were associated with the clinical improvement using Pearson’s correlation. A) Visualization of resulting streamlines associated with the optimal clinical outcome in an MNI152 NLin2009bAsym template space overlayed on the BigBrain template ^28^. B) Validation of DBS Fiber Filtering results for streamlines passing electric field values thresholded at different magnitues using circular (in-sample) and ten-fold analysis.

# Supplementary Results S2:

**Ridge regression model (additional results)**

In the joint model, DBS Fiber Filtering showed the largest standardized coefficient (*β* = 0.127), followed closely by DBS Sweet Spot (β = 0.116), structural DBS Network Mapping (*β* = 0.107), coordinate proximity (*β* = 0.075) and functional DBS Network Mapping (*β* = 0.072) (Figure 3B). Statistical significance of the estimates is typically not reported in ridge regression models due to the shrinkage factor, that influences the ß estimates determined by the model.

Our joint model explained 12% of the variance in clinical outcomes in the heterogeneous, group-level validation cohort. As indicated by the coefficient of determination, the variance explained by fitting a general linear model to surrogate variables from individual methods on the validation cohort was lower in all instances (*R^2^* proximity = 0.037; *R^2^* DBS Sweet Spot = 0.065; *R^2^* DBS Fiber Filtering = 0.036; *R^2^* DBS Network Mapping SC = 0.092; *R^2^* DBS Network Mapping FC = 0.088).

Due to different clinical improvement scale of the prospectively acquired contact-wise stimulation cohort, calculating actual clinical predictions was not possible. However, we fitted general linear model for each method and correlated the estimates with the empirical clinical improvement (Supplementary Figure 9). For each method, we calculated the success rate (%) of matching the top empirical contact with the one selected by our model, as well as the empirical contact ranking being top 2 or top 3 contacts selected by our model. We also calculated the success rate (%) of our model in selecting the correct segment for beneficial stimulation (Supplementary Table 6).


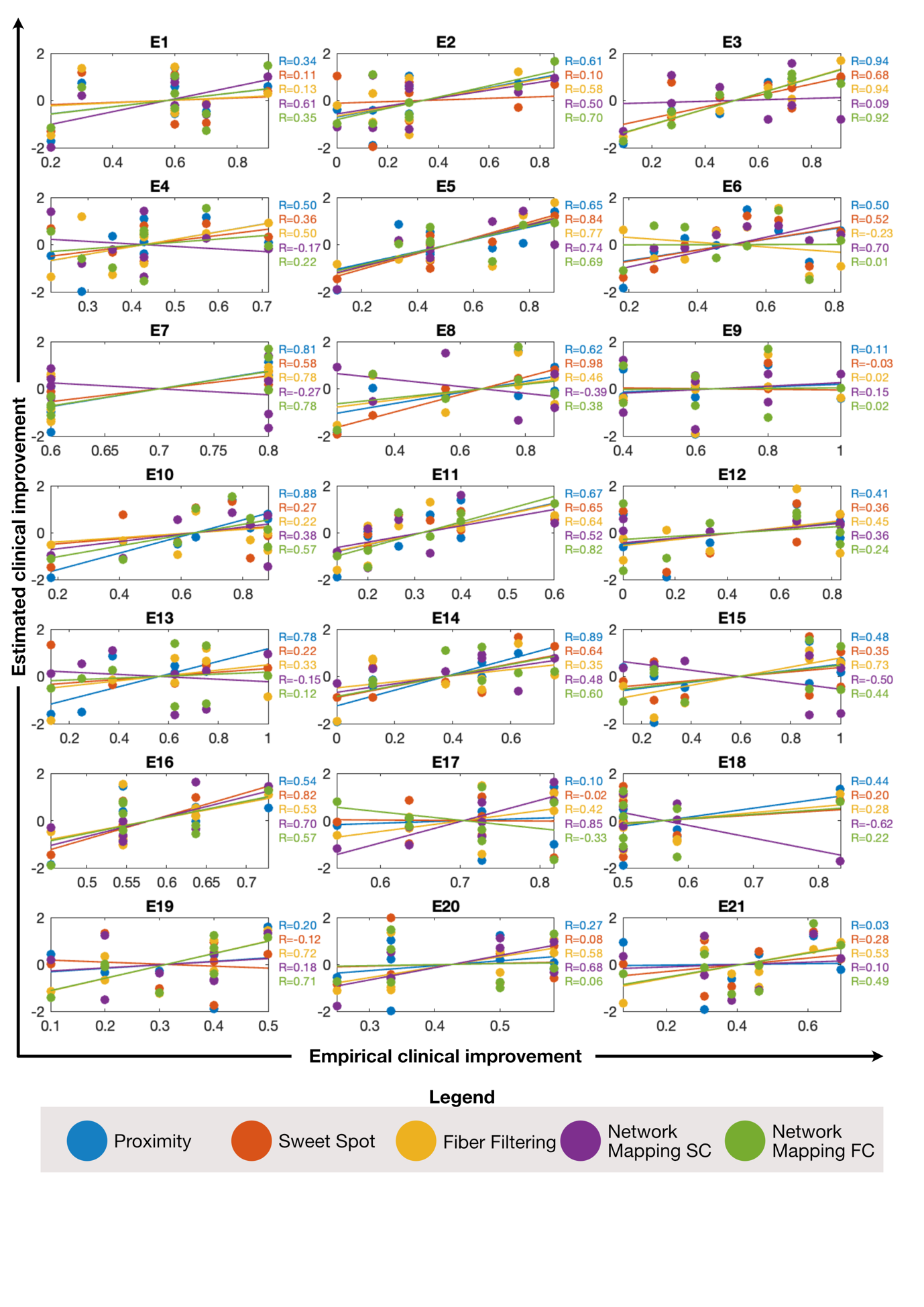


**Supplementary Figure S9. Estimates of clinical improvement in the contact-wise stimulation cohort using five different modelling methods.** Each model was trained on one independent dataset. A general linear model was then fitted to that dataset, and clinical improvements were estimated in the new contact-wise stimulation cohort. Estimated improvements for each method were normalized and plotted on the y-axis against the empirical clinical improvement associated with each electrode contact on the x-axis. Note that in some cases, multiple electrode contacts shared the same empirical improvement value.

**Supplementary Table S6. Individual model performance on contact-wise stimulation data.** The percentage (%) of matches between the optimal clinical contact and the model-selected top contact, top two contacts, top three contacts, as well as the agreement between the model-selected and clinically selected stimulation levels across different methods.

|  | **Top 1 Contacts (%)** | **Top 2 Contacts (%)** | **Top 3 Contacts (%)** | **Level**  **(%)** |
| --- | --- | --- | --- | --- |
| **Proximity** | 38 | 62 | 71 | 71 |
| **Sweet Spot** | 24 | 48 | 67 | 52 |
| **Fiber Filtering** | 19 | 57 | 81 | 52 |
| **Network Mapping SC** | 19 | 43 | 62 | 52 |
| **Network Mapping FC** | 29 | 52 | 67 | 57 |
| **Overall Model** | 33 | 67 | 86 | 71 |

**
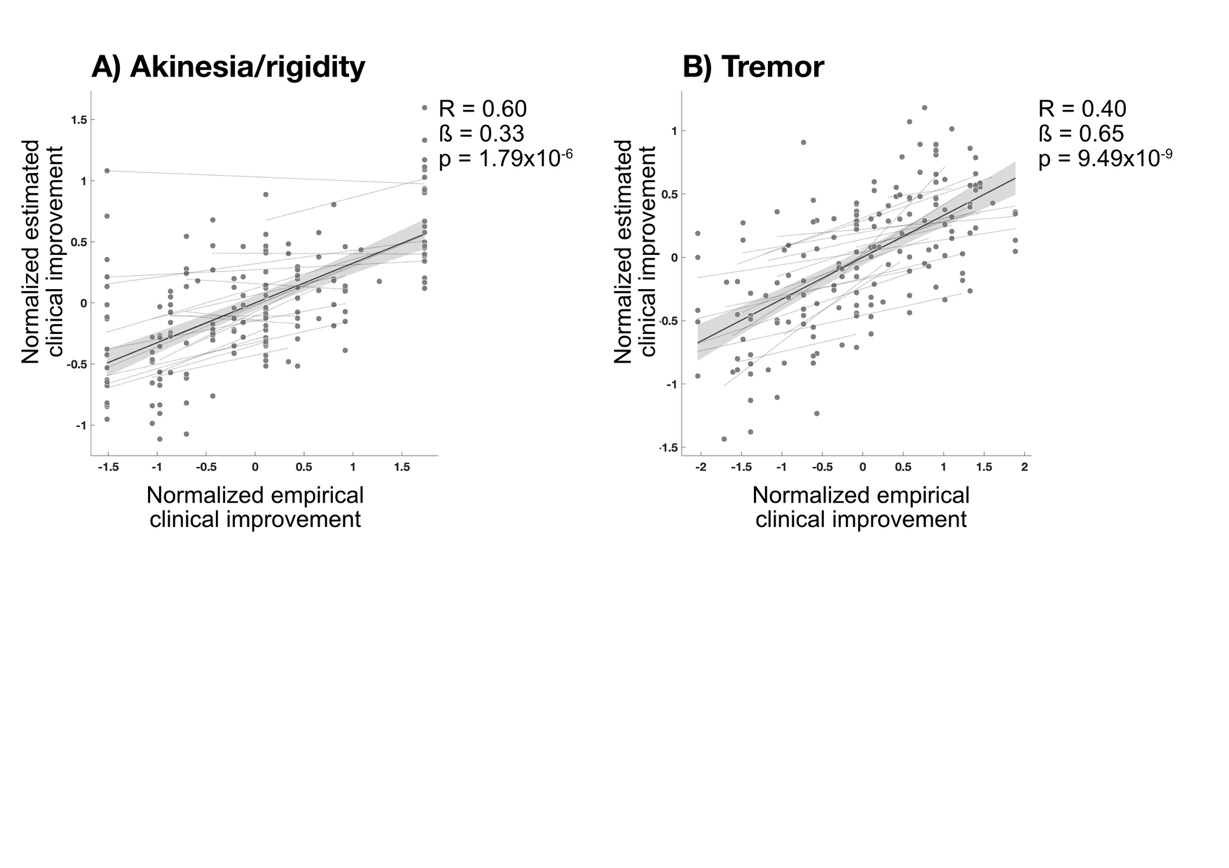
**

**Supplementary Figure S10. Linear mixed-effect model results for akinetic and tremor symptoms.** The combined ridge regression model was used to estimate clinical improvements for individual contacts specific to electrode placements in 21 cases. A linear mixed-effect model was fitted, with random intercepts for each individual electrode. A) Model-derived estimates of clinical improvement were positively correlated with the empirical improvements in akinesia. B) Model-derived estimates of clinical improvement were also positively correlated with the empirical improvement in tremor.

# Supplementary References

1. Cavallieri F, Fraix V, Bove F, et al. Predictors of Long-Term Outcome of Subthalamic Stimulation in Parkinson Disease. *Annals of Neurology*. 2020;89(3):587-597. doi:10.1002/ana.25994

2. Soulas T, Sultan S, Gurruchaga JM, Palfi S, Fénelon G. Depression and Coping as Predictors of Change After Deep Brain Stimulation in Parkinson’s Disease. *World Neurosurgery*. 2011;75(3):525-532. doi:10.1016/j.wneu.2010.06.015

3. Horn A, Reich M, Vorwerk J, et al. Connectivity Predicts deep brain stimulation outcome in Parkinson disease: DBS Outcome in PD. *Ann Neurol*. 2017;82(1):67-78. doi:10.1002/ana.24974

4. Alonso F, Zsigmond P, Wårdell K. Influence of Virchow-Robin spaces on the electric field distribution in subthalamic nucleus deep brain stimulation. *Clinical Neurology and Neurosurgery*. 2021;204:106596. doi:10.1016/j.clineuro.2021.106596

5. Muthuraman M, Deuschl G, Koirala N, Riedel C, Volkmann J, Groppa S. Effects of DBS in parkinsonian patients depend on the structural integrity of frontal cortex. *Sci Rep*. 2017;7(1):43571. doi:10.1038/srep43571

6. Richards M, Marder K, Cote L, Mayeux R. Interrater reliability of the unified Parkinson’s disease rating scale motor examination. *Movement Disorders*. 1994;9(1):89-91. doi:10.1002/mds.870090114

7. Bennett DA, Shannon KM, Beckett LA, Goetz CG, Wilson RS. Metric properties of nurses’ ratings of parkinsonian signs with a modified Unified Parkinson’s Disease Rating Scale. *Neurology*. 1997;49(6):1580-1587. doi:10.1212/WNL.49.6.1580

8. Siderowf A, McDermott M, Kieburtz K, et al. Test-Retest reliability of the Unified Parkinson’s Disease Rating Scale in patients with early Parkinson’s disease: Results from a multicenter clinical trial. *Mov Disord*. 2002;17(4):758-763. doi:10.1002/mds.10011

9. Metman LV, Myre B, Verwey N, et al. Test-retest reliability of UPDRS-III, dyskinesia scales, and timed motor tests in patients with advanced Parkinson’s disease: An argument against multiple baseline assessments. *Mov Disord*. 2004;19(9):1079-1084. doi:10.1002/mds.20101

10. Maks CB, Butson CR, Walter BL, Vitek JL, McIntyre CC. Deep brain stimulation activation volumes and their association with neurophysiological mapping and therapeutic outcomes. *J Neurol Neurosurg Psychiatry*. 2009;80(6):659-666. doi:10.1136/jnnp.2007.126219

11. Noecker AM, Frankemolle-Gilbert AM, Howell B, et al. StimVision v2: Examples and Applications in Subthalamic Deep Brain Stimulation for Parkinson’s Disease. *Neuromodulation*. 2021;24(2):248-258. doi:10.1111/ner.13350

12. Rajamani N, Friedrich H, Butenko K, et al. Deep brain stimulation of symptom-specific networks in Parkinson’s disease. *Nat Commun*. 2024;15(1):4662. doi:10.1038/s41467-024-48731-1

13. Neudorfer C, Butenko K, Oxenford S, et al. Lead-DBS v3.0: Mapping deep brain stimulation effects to local anatomy and global networks. *NeuroImage*. 2023;268:119862. doi:10.1016/j.neuroimage.2023.119862

14. Meyer GM, Hollunder B, Li N, et al. Deep Brain Stimulation for Obsessive-Compulsive Disorder: Optimal Stimulation Sites. *Biological Psychiatry*. 2024;96(2):101-113. doi:10.1016/j.biopsych.2023.12.010

15. Horn A, Li N, Dembek TA, et al. Lead-DBS v2: Towards a comprehensive pipeline for deep brain stimulation imaging. *NeuroImage*. 2019;184:293-316. doi:10.1016/j.neuroimage.2018.08.068

16. Sobesky L, Goede L, Odekerken VJJ, et al. Subthalamic and pallidal deep brain stimulation: are we modulating the same network? *Brain*. 2022;145(1):251-262. doi:10.1093/brain/awab258

17. Butenko K, Neudorfer C, Dembek TA, et al. Engaging dystonia networks with subthalamic stimulation. *Proceedings of the National Academy of Sciences*. 2025;122(2):e2417617122. doi:10.1073/pnas.2417617122

18. Dembek TA, Roediger J, Horn A, et al. Probabilistic sweet spots predict motor outcome for deep brain stimulation in Parkinson disease. *Annals of Neurology*. 2019;86(4):527-538. doi:10.1002/ana.25567

19. Hollunder B, Ostrem JL, Sahin IA, et al. Mapping dysfunctional circuits in the frontal cortex using deep brain stimulation. *Nat Neurosci*. 2024;27(3):573-586. doi:10.1038/s41593-024-01570-1

20. Rajamani N, Friedrich H, Butenko K, et al. Deep brain stimulation of symptom-specific networks in Parkinson’s disease. *Nat Commun*. 2024;15(1):4662. doi:10.1038/s41467-024-48731-1

21. Åström M, Diczfalusy E, Martens H, Wårdell K. Relationship between neural activation and electric field distribution during deep brain stimulation. *IEEE Trans Biomed Eng*. 2015;62(2):664-672. doi:10.1109/TBME.2014.2363494

22. Van Essen DC, Ugurbil K, Auerbach E, et al. The Human Connectome Project: A data acquisition perspective. *Neuroimage*. 2012;62(4):2222-2231. doi:10.1016/j.neuroimage.2012.02.018

23. Goede LL, Al-Fatly B, Li N, et al. Convergent mapping of a tremor treatment network. *Nat Commun*. 2025;16(1):4772. doi:10.1038/s41467-025-60089-6

24. Ríos AS, Oxenford S, Neudorfer C, et al. Optimal deep brain stimulation sites and networks for stimulation of the fornix in Alzheimer’s disease. *Nat Commun*. 2022;13(1):7707. doi:10.1038/s41467-022-34510-3

25. Horn A, Reich MM, Ewert S, et al. Optimal deep brain stimulation sites and networks for cervical vs. generalized dystonia. *Proc Natl Acad Sci USA*. 2022;119(14):e2114985119. doi:10.1073/pnas.2114985119

26. van der Linden C, Berger T, Brandt GA, et al. Accelerometric Classification of Resting and Postural Tremor Amplitude. *Sensors (Basel)*. 2023;23(20):8621. doi:10.3390/s23208621

27. Ewert S, Plettig P, Li N, et al. Toward defining deep brain stimulation targets in MNI space: A subcortical atlas based on multimodal MRI, histology and structural connectivity. *NeuroImage*. 2018;170:271-282. doi:10.1016/j.neuroimage.2017.05.015

28. Amunts K, Lepage C, Borgeat L, et al. BigBrain: An Ultrahigh-Resolution 3D Human Brain Model. *Science*. 2013;340(6139):1472-1475. doi:10.1126/science.1235381

29. Caire F, Ranoux D, Guehl D, Burbaud P, Cuny E. A systematic review of studies on anatomical position of electrode contacts used for chronic subthalamic stimulation in Parkinson&apos;s disease. *Acta Neurochir (Wien)*. 2013;155(9):1647-54-discussion 1654. doi:10.1007/s00701-013-1782-1

30. Horn A, Kühn AA, Merkl A, Shih L, Alterman R, Fox M. Probabilistic conversion of neurosurgical DBS electrode coordinates into MNI space. *NeuroImage*. 2017;150:395-404. doi:10.1016/j.neuroimage.2017.02.004

31. Bejjani BP, Dormont D, Pidoux B, et al. Bilateral subthalamic stimulation for Parkinson’s disease by using three-dimensional stereotactic magnetic resonance imaging and electrophysiological guidance. *Journal of Neurosurgery*. 2000;92(4):615-625. doi:10.3171/jns.2000.92.4.0615

32. Akram H, Sotiropoulos SN, Jbabdi S, et al. Subthalamic deep brain stimulation sweet spots and hyperdirect cortical connectivity in Parkinson&apos;s disease. *NeuroImage*. 2017;158:332-345. doi:10.1016/j.neuroimage.2017.07.012

33. Bot M, Schuurman PR, Odekerken VJJ, et al. Deep brain stimulation for Parkinson&apos;s disease: defining the optimal location within the subthalamic nucleus. *J Neurol Neurosurg Psychiatr*. Published online January 20, 2018:jnnp-2017-316907-7. doi:10.1136/jnnp-2017-316907

34. Horn A. The impact of modern-day neuroimaging on the field of deep brain stimulation. *Current Opinion in Neurology*. 2019;32(4):511-520. doi:10.1097/WCO.0000000000000679

35. Edlow BL, Mareyam A, Horn A, et al. 7 Tesla MRI of the ex vivo human brain at 100 micron resolution. *Sci Data*. 2019;6(1):244. doi:10.1038/s41597-019-0254-8
